# Clinical and operational insights from data-driven care pathway mapping: a systematic review

**DOI:** 10.1186/s12911-022-01756-2

**Published:** 2022-02-17

**Authors:** Matthew Manktelow, Aleeha Iftikhar, Magda Bucholc, Michael McCann, Maurice O’Kane

**Affiliations:** 1 Centre for Personalised Medicine, Clinical Decision Making and Patient Safety, Ulster University, C-TRIC, Altnagelvin Hospital Site, Derry-Londonderry, Northern Ireland; 2 School of Computing, Engineering and Intelligent Systems, Ulster University, Magee, Derry-Londonderry, Northern Ireland; 3 Department of Computing, Letterkenny Institute of Technology, Co. Donegal, Ireland; 4 Clinical Chemistry Laboratory, Altnagelvin Hospital, Western Health and Social Care Trust, Derry-Londonderry, Northern Ireland

**Keywords:** Care pathway, Clinical workflow, Clinical pathway, Process mining, Electronic Records, Review

## Abstract

**Background:**

Accumulated electronic data from a wide variety of clinical settings has been processed using a range of informatics methods to determine the sequence of care activities experienced by patients. The “as is” or “de facto” care pathways derived can be analysed together with other data to yield clinical and operational information. It seems likely that the needs of both health systems and patients will lead to increasing application of such analyses. A comprehensive review of the literature is presented, with a focus on the study context, types of analysis undertaken, and the utility of the information gained.

**Methods:**

A systematic review was conducted of literature abstracting sequential patient care activities (“de facto” care pathways) from care records. Broad coverage was achieved by initial screening of a Scopus search term, followed by screening of citations (forward snowball) and references (backwards snowball). Previous reviews of related topics were also considered. Studies were initially classified according to the perspective captured in the derived pathways. Concept matrices were then derived, classifying studies according to additional data used and subsequent analysis undertaken, with regard for the clinical domain examined and the knowledge gleaned.

**Results:**

254 publications were identified. The majority (n = 217) of these studies derived care pathways from data of an administrative/clinical type. 80% (n = 173) applied further analytical techniques, while 60% (n = 131) combined care pathways with enhancing data to gain insight into care processes.

**Discussion:**

Classification of the objectives, analyses and complementary data used in data-driven care pathway mapping illustrates areas of greater and lesser focus in the literature. The increasing tendency for these methods to find practical application in service redesign is explored across the variety of contexts and research questions identified. A limitation of our approach is that the topic is broad, limiting discussion of methodological issues.

**Conclusion:**

This review indicates that methods utilising data-driven determination of de facto patient care pathways can provide empirical information relevant to healthcare planning, management, and practice. It is clear that despite the number of publications found the topic reviewed is still in its infancy**.**

**Supplementary Information:**

The online version contains supplementary material available at 10.1186/s12911-022-01756-2.

## Background

### Overview

In very many healthcare systems around the world, patient care guidance in medical practice is implemented through clinical care pathways [1]. Defined as “complex interventions for the mutual decision making and organisation of care processes for a well-defined group of patients during a well-defined period” [2], the benefits espoused for their application include better patient outcomes and cost savings arising from operational efficiencies [3]. Similar positive outcomes are also proposed for the deployment of electronic health records [4]; together these advances comprise a useful framework for the implementation of evidence-based medicine (EBM). However, there exists a tension between the imperative to best deliver patient-centred care and the necessity for guidance to be clear, memorable, and easily interpretable by clinicians under pressure. Time spent interacting with the electronic record and referencing guidance is necessarily time not spent meaningfully interacting with the patient, but if a care pathway does not take account of a patient’s clinical history and circumstances it will not support personalisation of care. Guidance providers such as UK National Institute for Clinical Excellence (NICE) attempt to strike a balance, using health economic assessments based on the available data to classify for which patients a treatment is appropriate. The quality of this effort however can only be as good as the evidence base, and in the absence of specific studies on the applicability of treatment to particular patient groups, assumptions of statistical homogeneity in clinical trials mean that, to quote de Leon: “the current status of RCTs is that they can tell us which treatments are effective but not necessarily which patient should receive them” [5]. Particularly in patients with multimorbidity the risks and benefits of treatments may differ [6, 7], and the pressure to practice “defensive medicine” in an increasingly litigious environment [8] and a lack of resources to undertake labour-intensive rationalisation of treatment plans [9] compound this problem. The case has also been made that currently defined clinical care pathways may need to be substantially restructured, to take advantage of new diagnostic technologies [10].

Responses to these challenges must take into account that a clinical care pathway as defined in [2] above is the “should-be” or formal pathway, a somewhat idealised construct intended to appropriately guide the patient care journey to achieve consistent best practice and optimal patient flow. Variations might arise from this defined pathway appropriately due to clinical acumen or patient complexity, or otherwise through unforeseen circumstances, organisational care boundaries, or deviations from guidance. In practice, the sequence of care processes experienced by a cohort of patients comprises a set of “de facto” pathways [11], corresponding in varying degree to the formally defined care pathway. Latterly, there has been increasing interest in applying algorithmic methods to accumulated electronic patient care data to determine these “de facto” care pathways.

### Patient care process discovery

Patient care processes are generally considered particularly challenging to describe and model in a realistic and comprehensive fashion. Methodologies for analysing and describing processes have often been derived from manufacturing or service industries; where the analysis proceeds from routinely collected data, the procedure used is often referred to as “process mining”. In such contexts, both the environment and the sequence of unit operations performed in the process are highly structured. Clinical care is likewise delivered in a highly structured environment, but also one that is highly dynamic and extremely complex [12]. The sequence of unit operations performed is often only partially defined, with certain sets of activities following absolute sequencing requirements (for example, anaesthesia must precede surgery), but which may be scheduled on an ad hoc basis according to the intervention of a clinician. Other activities such as general nursing care or routine observations may take place on a schedule unrelated to other activities. The inherent diversity of patients and hence care processes adds a further level of complexity to the picture; which may finally be compounded by variability in the quality of the available data, in terms of its granularity, accuracy, and completeness.

In response to the challenge of interpreting this complex and often incomplete data pool, a broadly similar general procedure is followed. A particular data source is identified, which records some aspect of activities relating to clinical care. Depending on the environment and particular healthcare process being examined, the data may require substantial processing before it is suitable for use, unless it was collected solely for research or audit purposes: generally, data quality and completeness is a key issue. The types of data that are available for use depend very strongly on the context being explored, but may include include whole or filtered electronic health records, used primarily for clinical care; registries capturing care pathway information along with clinical data; or administrative data recorded from Hospital Information Systems, such as Patient Administration Systems or systems used to generate insurance billing reports. Only structured data can be directly interpreted; Wang et al. [13] review the active research topic of clinical information extraction, where Natural Language Processing (NLP) facilitates the automatic extraction of concepts, entities, events, and their associations from the unstructured free text commonplace in electronic health records.

Temporal data may be present (for example, timestamps), or it may be implicit (for example, the sequence of recorded activities). The data may be filtered for relevance, sometimes drastically, or simplified, for example by aggregating synonyms or abstracting patterns.

The system is then described from the data, generally in the form of an algorithmically derived “process model”, often represented as a network or connected graph of states and likelihood of transition between states. With the states representing care activities, this process model can be considered as a representation of a particular perspective on the aggregated de facto care pathways experienced by the patients in the dataset. The care pathways thus derived need not be linear in nature; iterative or cyclical pathways are common in many clinical domains. The process model often does not describe the entire data set, but only those possible paths through the states with sufficient “support” from the data. Often, some degree of clustering of the data is performed so that similar paths are merged in a consensus path with support from the variations. How tightly defined a process is and the quality of the data collected on it determines the extent of filtering and clustering required. In some cases the majority of the dataset is discarded, and in others all data is incorporated into the model. The steps involved in preprocessing the data may be revisited during the construction of the process model, or temporal data extracted at an earlier point may be utilised in the construction of the process model.

### Review rationale

Determination of de facto care pathways derived from accumulated electronic data has clear potential to enhance understanding of clinical services. To address the complex challenges outlined in the “Overview” section above, it is likely that further methods of analysis and additional data will need to be utilised in combination with the derived care pathways. Furthermore, assessments of the utility in practice of methods deriving and analysing de facto care pathways will be required. While these topics are frequently present in the research literature, we are unaware of any previous review which has considered these questions in depth. We thus undertook a comprehensive and systematic review of the literature in accordance with the PRISMA 2020 key reporting guideline [14], with such elaborations as were necessary due to the intersectional and evolving nature of the topic.

### Objectives

The literature being considered undertakes methodologically complex analysis of observational data to derive quantitative representations of practice, which are however often evaluated qualitatively. Comparison of practice across different settings may be presented, but outcomes compared may not be readily translatable for comparison with other studies given the variety of contexts and metrics possible. As such, utilising the PICOS framework endorsed by PRISMA for interventional studies would be unlikely to yield useful results, and we instead develop our review questions with reference to the Population—Phenomena of Interest—Context -Type of Studies (PPCT) framework developed by the Joanna Briggs Institute for reviews of mixed methods studies [15].

In literature identifying as carrying out process mining, the set of derived care pathways would be described as a process model, and the frame of reference as a perspective [16]. We follow this terminology, though we do not restrict our review to literature that does so.

With regard to the characteristics of the literature of interest described in Table 1, we therefore define the following review questions:

- **Review Question 1:** What are the main characteristics of the identified literature in terms of year of publication, clinical specialism considered, and country of origin of dataset?
- **Review Question 2:** The de facto care pathways experienced by patients might be defined from the perspective solely of their clinical context; of the healthcare practitioner undertaking their care; the location care is performed; or as care activities capturing some combination of these aspects of care, henceforth the “administrative/clinical” perspective. To what extent does the identified literature reflect these different perspectives?
- **Review Question 3:** What are the main characteristics of the literature in terms of application of further analysis to the derived de facto pathways, with or without integration with additional patient-related data?
- **Review Question 4:** To what extent and how has process mining of de facto care pathways shown practical utility?

**Table 1:** Review definition following the PPCT framework [15]

PPCT framework item	Definition
Population	Real patients who have undergone clinical care whose electronic data captures some aspect of care related activities
Phenomena of interest	The abstraction of sequential care activities from that data to derive a set of de facto care pathways; any use of additional techniques or data facilitating further evaluation of the derived de facto pathways; and any assessments of the practical utility of the research in the context from which the data derived
Context	The sequential care activities described above are undertaken on patients with evolving clinical context, carried out by particular clinical roles, and may take place in a sequence of specific locations. The de facto care pathways experienced by patients may be defined from the frame of reference of any of these aspects of clinical care
Types of studies	All reports where some discussion of the relevance of the derived care pathways takes place, therefore excluding the use of synthetic data or purely methodological reports, but including different analyses on the same study

We treat Review question 1 quantitatively; undertake classifications of the literature to answer Review questions 2 and 3; and treat Review question 4 primarily in a narrative fashion.

## Methods

### Identification of search strategy

Prior to initiating the search, we were aware of some literature that we considered of interest [17–21] and of the definitive 2016 review on the subject of process mining in healthcare by Rojas et al. [22]. Comparison of the literature of interest with that review indicated some variation in terminology used, particularly when considering terms present in the title, abstract, and keywords, the searchable content of curated indexed literature databases.

We considered that the apparent variation in terminology used in the literature was characteristic of an emerging intersectional topic, and might be partly attributable to conceptually similar research being reported from different perspectives in journals from very different disciplines. For example, medical specialty journals might focus on the applicability of the methods applied in their clinical context, while computer science journals might place greater emphasis on the specifics of the implementation or the advances in methodology developed. Given this variation in terminology, exploratory efforts to construct search terms adequately capturing the diversity of the literature were only partly successful, and as we felt it would be inappropriate to inadvertently restrict the search to a particular domain the need for a modified approach to literature search was apparent.

It has been proposed by Greenhalgh and Peacock [23] that in systematic reviews of complex or heterogeneous evidence in the field of health services research, “snowball” methods of forward (citation) and backwards (reference) searching are especially powerful. The approach is likewise recommended for systematic searches of information systems literature [24] and is referred to in PRISMA 2020 [14]. Preliminary experimentation with this methodology yielded positive results: it was therefore developed as presented below.

### Search strategy

The search strategy comprised the following tasks:

- **Task 1:** Construction of a suitable search term.
- **Task 2:** Identification of the optimal information source by application of the search term to a variety of literature databases.
- **Task 3:** Accumulation of an initial screened publication set from the selected database.
- **Task 4:** Screening of literature citing the initial publication set (forward search); removal of duplicates.
- **Task 5:** Filtering of referenced literature from the combined initial and forward search (backwards search) via a second search term, followed by manual screening.
- **Task 6:** Screening of literature identified in previous applicable reviews to identify any relevant publications not previously found, and screening of citations of that literature.
- **Task 7:** Search with the term constructed in Task 1 of a second database with different topic coverage.
- **Task 8:** Hand screening of relevant indexed journals not covered by the selected electronic database.

### Search term construction (Task 1)

We constructed our search term using concepts from the “Population” and “Phenomena of Interest” items of Table 1 above, with reference to the literature of interest already identified. The application of the “snowball” search methodology means the main requirement for the initial screened publication set is to achieve a broad representative sampling of the literature rather than complete coverage initially. In this case, the particular challenge is to capture relevant literature from across the data and process analytics communities.

We initially include alternative and related terms to “clinical pathway”, particularly those present in the literature we are already aware of. The other concept in the first part of the “Phenomena of Interest” item relates to algorithmic information extraction, covered by the term “mining”. The “Population” item is referenced by adding the term “electronic record”. Since we wish to screen a wide variety of literature for our initial search, “mining” and “electronic record” are combined as alternatives rather than being required to both be present. The search term to be applied in tasks 2 and 3 of the search strategy is thus:

S1: ( "clinical pathway*" OR "critical pathway*" OR "care pathway*" OR "clinical workflow" OR "careflow" ) AND ( "electronic record" OR "mining" )

While the lack of synonyms for electronic record might be considered to risk only a portion of the relevant literature being captured, addition of further terms did not readily improve coverage. In any case we anticipated that the further stage of the search will achieve good coverage beyond any limitations of the initial stage.

As we anticipate a very large number of references to be identified in the backwards search (task 5 of the search strategy), a “filtering” search term is required. This filtering search term S2 is intended to remove methodological references not within the health informatics domain and is therefore based upon but less restrictive than the initial search term above:

S2: (“pathway*" OR "clinical workflow" OR "careflow").

### Eligibility criteria

With reference to the framework expressed in Table 1 above, we define the following inclusion and exclusion criteria:

- **Inclusion Criteria 1:** English language literature with available full text published after 2000. As the topic under examination is relatively recently established, no indexed content was excluded, for example book chapters and conference proceedings were included.
- **Inclusion Criteria 2:** As defined in the objectives above, literature involving the processing of a real (not synthetic) clinical dataset describing sequential activities relating the care of a set of patients to derive a representation of the care process that captures the variety of de facto care pathways experienced by patients. Initial rejection is on title and abstract, with inclusion after a further check of the full text.
- **Exclusion Criteria 1:** Literature where only very limited, extrapolated, or simulated patient data was used, or where the focus is exclusively methodological with no discussion of the derived de facto care pathways.
- **Exclusion Criteria 2**: Trials evaluating the effect of novel clinical interventions.

### Information sources

Suitable databases identified for evaluation in Task 2 were Dblp; Pubmed; Scopus; and Web of Science. Google Scholar was considered unsuitable as it does not offer a backwards (reference) search functionality.

Table 2 presents the results of applying the search term S1 to the identified databases; MM searched title, keywords, and abstract on 13th January 2020.

**Table 2:** Database search results

Database	Results
Dblp	0
PubMed	105
Scopus	257
Web of science	178

From the database search results above, it was clear that Scopus (Elsevier) was the most appropriate database on which to conduct Tasks 3 through 5 of the search strategy, particularly given its strong coverage in biomedical research [27]. In Scopus format, S1 is termed:

TITLE-ABS-KEY (("clinical pathway*" OR "critical pathway*" OR "care pathway*" OR "clinical workflow" OR "careflow") AND ( "electronic record" OR "mining")).

The secondary database used for Task 7 was selected to complement the focus of the primary database. For the purposes of selecting a database for Task 7 we considered the particular strength of Dblp to be computer science; Pubmed to be medical literature; and Scopus and Web of Science to be the life and physical sciences respectively. Given the absence of results from Dblp, for Task 7, Pubmed was identified as a database likely to have differing coverage to Scopus. In Pubmed format, S1 is termed:

("clinical pathway*"[All Fields] OR "critical pathway*"[All Fields] OR "care pathway*"[All Fields] OR "clinical workflow"[All Fields] OR "careflow"[All Fields]) AND ("electronic record"[All Fields] OR "mining"[All Fields]).

The initial search and screen (Task 3) was performed on 13th January 2020 by MM, and replicated by AI on 1st March 2021 with the search limited to publications between 1st January 2000 and 13th January 2020. The forward citation search and screen (Task 4) was performed initially by MM on 14th–15th January 2020, and by AI on 5th March 2021, again with the search limited to publications between 1st January 2000 and 13th January 2020. The reference filtering and screen was conducted by MM (Task 5) on 16th January 2020.

Previous reviews considered in Task 6 were identified throughout the search process and combined with those found through unstructured searches and incidentally. For Task 8, sources listed in the Index of Information Systems Journals [25] were manually screened by MM on title and website to identify relevant journals which might publish in the field of medical informatics and are not indexed by either the primary or secondary search databases.

### Study selection procedure

Literature identified in the initial search (Task 3) and forward citation search (Task 4) were screened independently on title, abstract and full text by two authors (MM and AI) in accordance with the eligibility criteria, with discrepancies resolved through consensus and a third author available in cases of disagreement. The filtered backward (reference) search (Task 5) was screened by MM primarily, with recourse to AI as needed.

Throughout Tasks 3–5, the inclusion and exclusion criteria were applied in a stepwise fashion, with initial screening conducted primarily on title and abstract with reference to the full text only in marginal cases. The intent was to not unduly restrict the search, as applying the exclusion criteria in this way allowed the references and citations of all literature initially appearing relevant to be assessed.

### Data extraction

Review Question 1: Following the framework outlined in the objectives, the date of publication, clinical domain, and country of origin of the dataset considered was extracted from the selected literature.

Review Question 2: The review question identified four possible frames of reference or perspectives from which derived care pathways might be constructed. If a study only considered care activities from the perspective of the responsible clinical role; location, whether physically or administratively assigned; or the clinical context of the patient, it was assigned to that perspective. Where the presentation and sequencing of care activities was not strictly limited to one of these perspectives, it was assigned to the administrative/clinical perspective most commonly considered in process mining in healthcare.

Review Questions 3 and 4: Literature applying supplemental techniques or utilising enhancing data was identified and held back for evaluation and classification during the data synthesis phase. Literature reporting practical utility for the results at any level of evidence was noted for narrative discussion after data synthesis.

All data extraction was carried out principally by MM, with recourse to AI and MOK as required to establish consensus.

### Data synthesis

As described in the Review Rationale, Objectives and Review Question 3, derived de facto care pathways may be subject to further analysis, henceforth “supplemental techniques”; or they may be “enhanced” with further data from the source dataset or otherwise [26].

Following Webster and Watson [24], the concepts of “supplemental techniques” and “enhancing data” as applied to derived care pathways were separated into units of analysis based on categorisations of these concepts constructed by two authors (MM in consultation with MOK) with regard to the relevant literature identified during the data extraction phase. The literature was then classified according to the units of analysis, deriving a “concept matrix”.

## Results

### Study selection

#### Primary search (Tasks 3–5)

Task 3 identified 257 publications for initial screening. Of these, 130 were retained after initial screening. A forward searches of citations yielded a further 120 relevant publications; a backwards search of references for those 250 publications yielded 49 further relevant publications after filtering and initial screening. 28 of those 299 publications were rejected due to unavailable full text, and a further 74 were rejected on full text screening. At the conclusion of Task 5, 197 publications had been selected.

#### Secondary search: screening and forward search of publications in other reviews (Task 6)

A literature review of varying extent is a common component of publications in this field. Through the primary search, incidental awareness, and ad hoc searches we located eleven previous relevant publications in which literature review is the primary motivation or component. We disregarded two of these [28, 29] as they appear to be conference publications preliminary to more comprehensive reviews published subsequently [22, 30]. Ghasemi and Amyot [31] conducted a systematised review; while they conducted a search to identify papers in the domain of process mining in healthcare, they did not screen these for relevance and rely on previous reviews for analysis of the published literature beyond simple demographics of the identified papers. The remaining eight reviews vary both in scope and methodology. Closest to the intent of this review is that of Yang and Su [32], where the focus is on process mining applications for clinical pathways. Unfortunately they do not detail their literature search methodology, so their criteria for inclusion and exclusion cannot be defined. Of the remainder, three focus on process mining in particular clinical areas. Kurniati et al. [33] focus on process mining in the single clinical domain of oncology; Williams et al. [34] conduct a general search of process mining in healthcare with the intent of reviewing those papers with at least a partial focus on primary care; while Farid et al. [35] restrict their search to process mining in the context of frail elderly care.

Riano and Ortega [36] focus on a broader class of computer technologies for medical treatment integration for management of multimorbidity; they include several examples of data and process mining under the descriptor “data integration”. Finally, three broader literature reviews of process mining in healthcare have been carried out by Rojas et al. [22]; Erdogan and Tarhan [30]; and Batista and Solanas [37], of which Rojas et al. is the most commonly cited. Table 3 summarises the review literature’s main attributes:

**Table 3:** Identified literature reviews of process mining in healthcare topics

References	Focus	Number of broader domain publications^a^ found	Fully referenced?	Number of publications reviewed
Yang and Su [32]	Clinical pathway process mining	37	Yes	37
Rojas et al. [22]	Process mining in healthcare	74	Yes	74
Ghasemi and Amyot [31]	Process mining in healthcare	168	No	3
Kurniati et al. [33]	Process mining in oncology	37	Yes	37
Erdogan and Tarhan [30]	Process mining in healthcare	172	Yes	172
Riano and Ortega [36]	Medical informatics for multimorbidity management	65 total; “data integration”, 16	Yes	“data integration”, 16
Williams et al. [34]	Process mining in primary care	143	Yes	7
Batista and Solanas [37]	Process mining in healthcare	55	Yes	55
Farid et al. [35]	Process mining in frail elderly care	8	Yes	8

^a^Domain publications refers to the broader domain assessed by the review in question, for example Williams et al. proceeded by initially searching for Process mining in healthcare, and screened for primary care

The referenced literature found in the nine reviews was screened for relevance and duplicates; the results of this screening are summarised in Table 4.

**Table 4:** Count of publications gleaned from previous reviews

References	Fully screened relevant available publications^a^
Yang and Su [32]	5
Rojas et al. [22]	14
Ghasemi and Amyot [31]	0
Kurniati et al. [33]	0
Erdogan and Tarhan [30]	1
Riano and Ortega [36]	3
Williams et al. [34]	20
Batista and Solanas [37]	0
Farid et al. [35]	0

^a^Screened in the order in which they appear in this table, therefore duplicate entries in more recent reviews will be excluded

Conducting a forward (citation) search on the 43 relevant publications using Google Scholar identified a further 14 relevant publications with full text available to us, yielding 57 new publications in total for this phase of the search.

#### Secondary search: PubMed search and hand screening of other journals (Tasks 7 and 8)

The 105 results of the PubMed search conducted on 13th January 2020 were screened by MM on 17th January 2020; no new relevant literature was found. Hand screening of literature in relevant journals listed in the Index of Information Systems Journals [25] but not indexed by PubMed or Scopus likewise yielded no new relevant literature.

#### Study selection summary

**Fig. 1:**
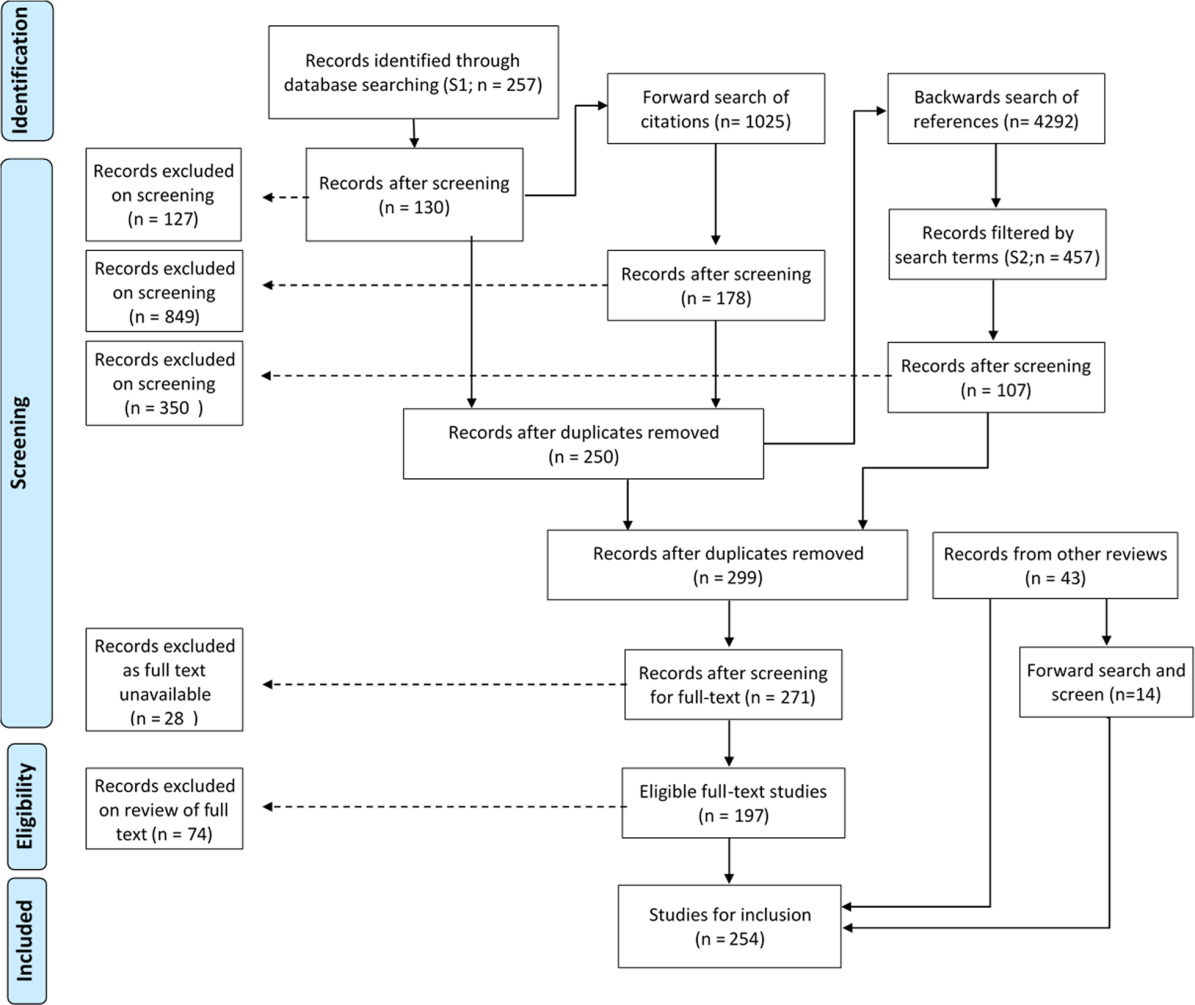
Following Moher D, Liberati A, Tetzlaff J, Altman DG, The PRISMA Group (2009). *P*referred *R*eporting *I*tems for *S*ystematic Reviews and *M*eta-*A*nalyses: The PRISMA Statement. PLoS Med 6(7):e1000097. 10.1371/journal.pmed1000097

Figure 1 below documents the search strategies that yielded relevant literature (Tasks 3–6). Tasks 3–5 identified 197 publications, while task 6 provided a further 57: totalling 254 publications deemed relevant.

### Characteristics of extracted data: Review Question 1

**Fig. 2:**
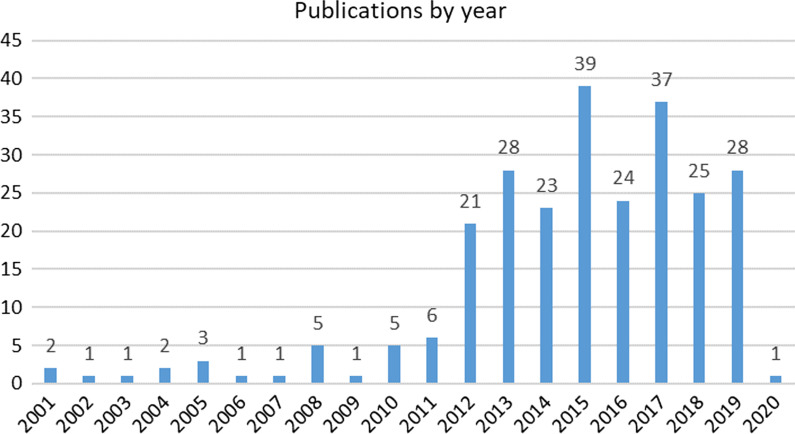
Identified literature by year of publication

**Fig. 3:**
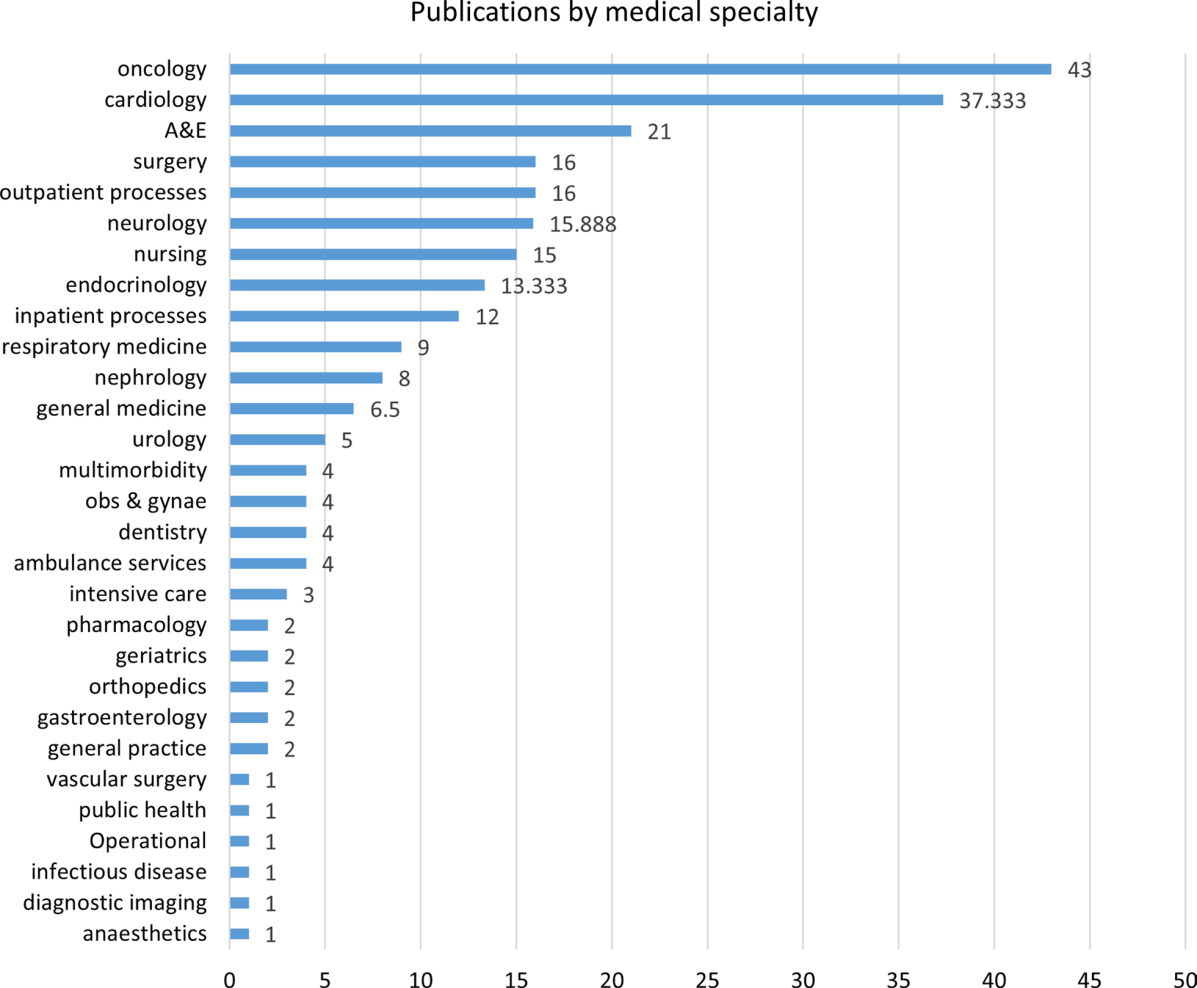
Identified literature by medical specialty

**Fig. 4:**
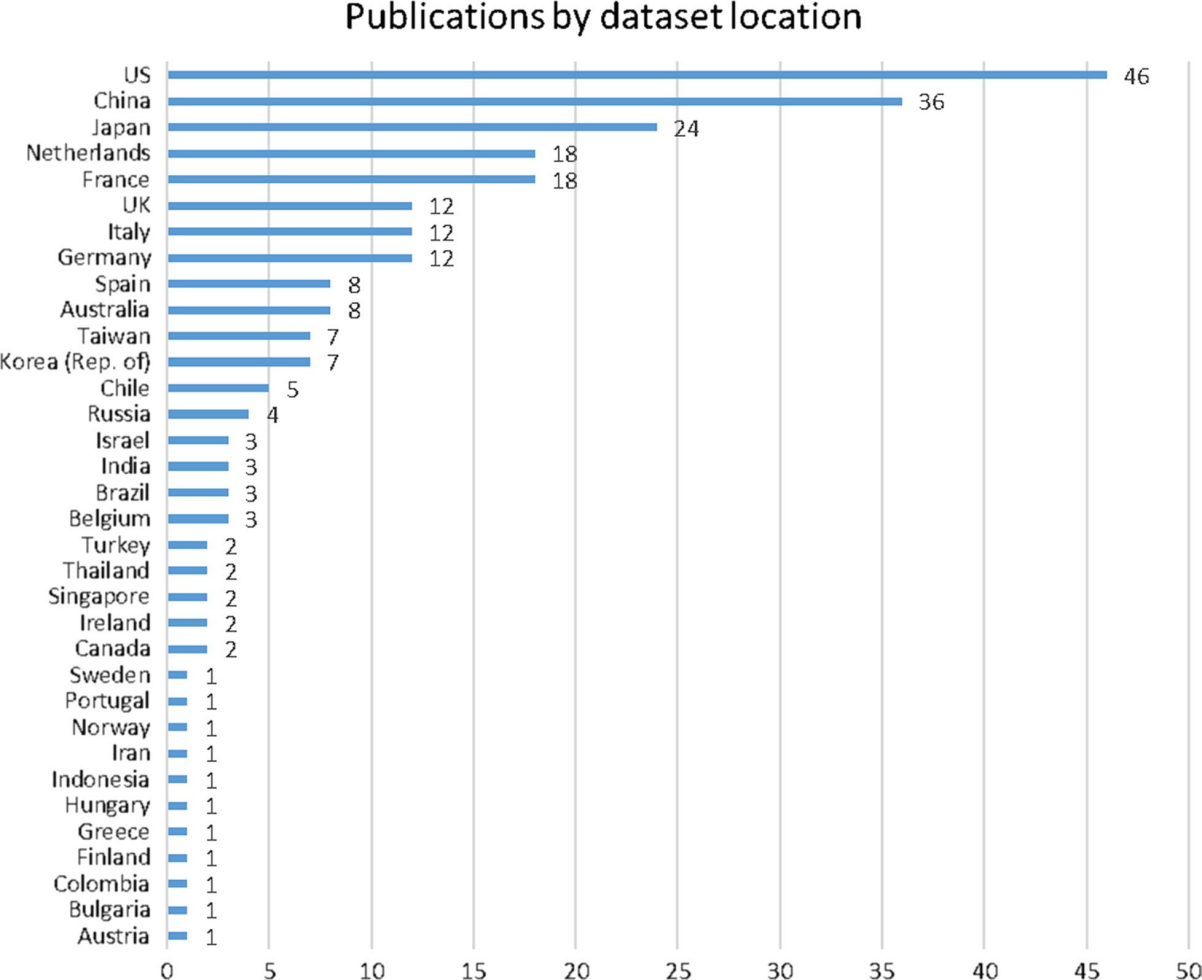
Identified literature by country of origin of dataset

Following data extraction performed as described in the “Data extraction” section, Figs. 2, 3 and 4 present the characteristics of the identified literature with regard to Review Question 1. Figure 4 classifies publications according to the country of origin of the healthcare data analysed, rather than by for example academic institution of the first author.

### Characteristics of extracted data: Review Question 2

Data extraction for Review Question 2 was conducted as described in “Data extraction” section above. Table 5 summarises the classification of literature identified in this review.

**Table 5:** Count of identified publications by care pathway perspective

Care pathway perspective	Examples of perspective	Number of publications
Administrative/clinical	Care activities may be for example nursing orders or clinic visits	217
Role Interaction	Care activities are described solely in terms of the healthcare practitioner performing them; transitions indicate referrals or handovers	5
Clinical context	Only clinical conditions or treatments relating to care activities are captured. A clinical task model restricted to a particular healthcare operation, for example sequential prescriptions or actions during a surgical operation, is also considered part of this classification	26
Location	Physical location, usually coded categorically	6

### Data synthesis, literature classification and narrative discussion: Review Questions 3 and 4

As described in the “Data synthesis” section, the “supplemental techniques” and “enhancing data” applied in further analysis of derived care pathways were identified as two separate units of analysis requiring categorisation. Working by consensus, examination of the literature identified during data extraction enabled construction of the category Tables 6 and 7 below.

**Table 6:** Identified categorisations of supplemental techniques

Enhancing data	Scope or definition
Outcomes	Clinical or other outcomes
Biomarkers	Clinical biomarkers; may be a biochemical marker or another disease or patient specific feature
Guidelines	Clinical or other formal guidelines
Comorbidities/complications	Where the care pathway perspective is not specifically disease based (“Clinical context” type, Table 5)
Prescriptions	Where the care pathway perspective is not specifically prescription based (“Clinical context” type, Table 5)
Clinical classification	For example, a triage score
Physical information	Geographical or local, where the care pathway perspective is not “location” as described in Table 5. Includes comparison of care processes across multiple sites
Other medical data	Ontologies of medical or surgical classification; treatment templates falling short of formal guidelines; comparator datasets; results from expert review panels

**Table 7:** Identified categorisations of enhancing data

Supplemental technique	Scope or definition
Clustering	Grouping of various derived care pathways from the derived model, usually based on some similarity measure; may include some comparative statistics of the different clusters. Distinguished from clustering carried out during production of the process model
Visualisation	Some means for the graphical display of derived or extracted care pathways beyond the usual process model. Usually implies the ability to select single or grouped patient care pathways for more detailed investigation
Statistical modelling	Either substantial analysis using simple descriptive or comparative statistics, or a more complex model derived using for example multilinear regression
Predictive modelling	The production and evaluation of a predictive model. Includes classification methods such as neural networks
Resource analysis	The analysis of the process model from the perspective of optimal resource allocation. Includes measures of efficiency and supplemental social network analysis
Conformance analysis	Assessment of the derived process model against guidance or expert opinion. Includes conformance against non-clinical requirements
Simulation/optimisation	Construction of a simulation model or optimisation of the process model against a particular metric

The remainder of the “Data synthesis, literature classification and narrative discussion: Review Questions 3 and 4” section is organised as follows:

- “Classification of care pathways derived from an Administrative/Clinical perspective: Review Question 3” section below discusses Review Question 3, presenting a classification of literature identified as deriving care pathways from the administrative/clinical perspective by combining the categories presented in Tables 6 and 7 in a modified “concept matrix” as described in “Data synthesis” section.
- Sections “Publications not utilising supplemental techniques” –“Statistical modelling” are organised according to the supplemental technique identified as being applied by the identified literature. Review Question 3 is illustrated by discussion of selected literature with reference to any enhancing data used. Review Question 4 is considered in a narrative fashion throughout by highlighting literature presenting “Outcomes” of practical utility for the results of their study.
- The “Care pathways derived from other perspectives” section and “Clinical context perspective” section consider Review Questions 3 and 4 in the context of the less common clinical context, location, and role interaction perspectives on care pathway derivation.

#### Classification of care pathways derived from an Administrative/Clinical perspective: Review Question 3

Table 8 presents a modified “concept matrix” [24], counting publications following the Administrative/Clinical perspective and presenting the numbers of publications found for each combination of supplementary techniques and enhancing data. This allows identification of areas of current interest, and highlights those areas where research is more sparse. The full table, showing references along with their clinical or other domain, can be found as Table A1 in Additional file 1: Appendix A (Additional file 1: Appendices A and B.docx).

**Table 8:** Count of publications deriving administrative/clinical care pathways, classified by supplemental technique and enhancing data used

	No supp. technique	Clustering	Visualisation	Statistical modelling	Predictive modelling	Resource analysis	Conformance analysis	Simulation/optimisation	Total
No enhancing data	23	18	4	1	9	13	15	3	86
Outcomes	2	2.83	3	2			2.5	2.5	14.83
Biomarkers	1	3.5	3		0.5				8
Guidelines	3	1.5	2	1	1	1	11.5	1	22
Comorbidities/complications		3.5	2	1	4				10.5
Prescriptions		*0.5*	2				1		3.5
Clinical classification	3	*2*	1.33	0.5	1	6		1.5	15.33
Physical information	4	3.33	2	0.5	1	1		7.5	19.33
Other medical data	8	2	1	3	5	3	12.5	3	37.5
Total	44	37.16	20.33	9	21.5	24	42.5	18.5	217

Obviously, some authors apply more than one technique, or use more than one type of enhancing data. Where the second technique or enhancing dataset is clearly subsidiary, we have identified the publication according to the main technique or enhancing dataset used. Where unavoidable we have duplicated entries, shown in italics in Appendix A, Table 1; for Table 8, these publications are counted fractionally

It is apparent that the substantial majority (89%; n = 194) of the 217 publications categorised utilise one or both of a supplementary technique or enhancing data. Supplementary techniques are more popular (80%; n = 173) than enhancing data (60%; n = 131). 51% of the total (n = 110) use both a supplementary technique and enhancing data. While variable, there is no trend in these proportions over time.

What is clear is that certain supplementary techniques have been much more frequently applied than others. The extent to which supplementary techniques have been combined with different types of enhancing data also varies quite substantially. If we consider the two most commonly applied techniques, conformance analysis and clustering, only 3.5 of the 42.5 publications using conformance analysis techniques utilise supplementary data other than “guidelines” or “other medical data”; while the supplemental technique of clustering has been applied to every type of enhancing data. While initially surprising, this disparity in the use of enhancing data can be explained if we consider the context in which supplementary techniques are used. Resource analysis, conformance analysis, and to a somewhat lesser extent simulation/optimisation are directly concerned with how clinical care is delivered in practice. As such, they tend to utilise enhancing data which directly constrain or determine practice (for example, guidelines or physical locations), or are at a higher level of abstraction (for example, a clinical classification such as a triage code might make reference to biomarker values and comorbidities).

We shall consider how supplementary techniques have been used with and without enhancing data in greater detail in the “Conformance analysis”–“Statistical modelling” sections below, using some brief descriptions of particular publications alongside summary tables describing example publications. Firstly however, in the “Publications not utilising supplemental techniques” section we consider those publications where a supplemental technique has not been used. Further information on some literature in these sections can be found in Additional file 1: Appendix B (Additional file 1: Appendices A and B.doc).

##### Publications not utilising supplemental techniques

If we first consider those publications not substantially utilising further techniques or significant enhancing data, these generally tend to draw on three motivations. Firstly, there are those publications in which a software package is applied to a dataset, and notable aspects of the derived process model are discussed without substantial use of supplemental techniques. In this case, the derived process map is considered of sufficient interest. Secondly, there are those publications where the results presented are explicitly preliminary to the application of supplemental techniques as further research. Finally, there are those publications where a novel method is presented or further developed, and the focus is on the accuracy of the derived process map rather than on the specific results obtained.

Publications which utilise enhancing data, but which we do not consider to apply supplemental techniques, tend to be of two types. In the first type, the derived care pathways are compared against the enhancing data, or the enhancing data partitions the process models. In the second approach, the enhancing data is incorporated into the process model.

Some examples of these various types of study are tabulated in Table 9 below.

**Table 9:** Illustrative examples of publications not utilising supplemental techniques

References	Notable for
Williams et al. [38] Le et al. [39]	Methodological focus. [38] evaluates different methodologies for dealing with incorrect sequencing in recorded data. [39] demonstrates a method for adding noise to records for analysis to enhance privacy
Prodel et al. [40]	Methodological focus, preliminary to further research, with discussion of clinical relevance of derived pathways. Methodology claims to reconstruct patient pathways from recorded data with optimal information content and improved computational efficiency; complication, readmission, and mortality data derived for different pathways; derived pathways and outcomes intended to be translated into formalisms suitable for direct use in simulation
Uragaki et al. [41] Williams et al. [42] Mans et al. [43] Partington et al. [44]	Enhancing data used for comparison against derived care pathways. Derived pathways are compared against expert consensus in [41], identifying substantial variation for non-pharmacological interventions. [42] considers prescribing practice and adverse events with regard to specific guidelines. [43, 44] compare derived pathways at multiple different sites, identifying or confirming variations in clinical practice
Baker et al. [19]	Enhancing data incorporated into the process model. Comprehensive Markov model developed from clinical records, providing detailed picture of frequency and context of complications. Explicitly intended to be similar to model used in health economics, facilitating future health technology assessment

##### Conformance analysis

Conformance analysis in the context of process mining was defined by Van der Aalst in 2011 [45] as one of the three main forms of process mining, utilised where both a pre-existing “process model” and an event log are available. In the particular context of healthcare, the pre-existing “process model” is often a protocol, guideline, or formally defined care pathway, and electronic care records generally take the role of the event log. It is the most commonly applied supplemental technique among the collated publications, although as noted above it has not been frequently combined with enhancing data other than those mentioned. The recent deployment in clinical practice in two hospital settings of the pMineR R library [46] seems likely to further facilitate and encourage this type of analysis. Table 10 below tabulates some indicative literature utilising conformance analysis.

**Table 10:** Illustrative examples of publications undertaking conformance analysis on derived de facto patient pathways

References	Notable for
Lenkowicz et al. [47]	Application of pMineR library to conformance analysis of translated clinical guidelines
Poelmans et al. [48]	Identification of quality of care issues at individual and group levels; subsets of patients with more complex care needs and pathways; and requirement for redesign of formal care pathway
Li et al. [49]	Determination of odds-ratios for the effect on outcomes of a variation in practice
Hwang et al. [50] Yang and Hwang [51]	Detection of non-standard clinical practice identifying fraudulent reimbursement claims
Bouarfa and Dankelman [52]	Outlying practices in laparoscopic surgery workflows identified from video-derived physical position process model

##### Clustering and visualisation

While they are separate techniques, we shall consider clustering and visualisation together. A distinction can be drawn between those publications where clustering or visualisation is undertaken as a means towards a human-interpretable process overview; and those publications in which clustering or visualisation facilitates investigation of the relationship between approximated models of derived care pathways and enhancing data.

In the first case, visualisation (for example, [53, 54]) or, less frequently, clustering (for example, [55]) has been used to render tractable the investigation of very large datasets. This is not to say that such methods are a requirement for dealing with large datasets; Ainsworth and Buchan [56] applied conformance analysis to an administrative/clinical process model of 100,000 Chronic Kidney Disease (CKD) patients extracted from the Salford Integrated Record, without utilising clustering techniques. Nor does a dataset need to be particularly large for visualisation to be a useful tool; Hirano and Tsumoto [57, 58] visualised physical movements in an administrative/clinical process model for 3443 outpatients of Shimane University Hospital, Japan, and Klimov et al. [59] abstracted clinical biomarkers and other data for visualisation for a dataset of more than 1000 patients of the University of Chicago Bone Marrow Transplantation Centre.

Regarding the second case, certain publications apply a visualisation toolbox to present real patient pathways, filtered or otherwise arranged by patient characteristics such as biomarkers. Table 11 below tabulates some examples of these types of studies.

**Table 11:** Examples of publications utilising clustering or visualisation as a supplemental technique

References	Notable for
Basole et al. [60, 61], utilizing toolbox of Kumar et al. [62] Bettencourt-Silva et al. [63, 64]	Visualisation of patient pathways filtered and/or aggregated according to biomarkers and clinical characteristics
Caballero et al. [65]	Combine visualisation of biomarkers and conformance analysis against guidelines across patient derived care pathways
Ozkaynak et al. [66]	Variations in workflow according to triage acuity across multiple sites determined using transition matrix representations of visualised derived care pathways
Perer et al. [67] Huang et al. [68]	Sankey diagrams used to present association of care pathways with prescriptions [67] and comorbidities and complications [68]
Zhang and Padman [69] Zhang et al. [70] Dagliati et al. [18, 71] Najjar et al. [72] Nuemi et al. [73]	Representative care pathways visualised from clustering derived care pathways. Enhanced with comorbidity data [70, 72], correlated with biomarkers [18, 71], or across multiple sites [73]

##### Predictive modelling

Predictive modelling has been relatively frequently and widely applied. The absence of predictive modelling techniques utilising outcome or prescription data can be explained by such studies tending to focus on a clinical context perspective, being based on sequences of health or treatment states rather than the administrative/clinical activities categorised above.

Frequently, predictive modelling of derived care pathways is undertaken to develop tools to support or enhance clinical decision making, through the provision of for example a differential diagnosis [74] or predicted workflow steps [75]. A multiplicity of methods exist, but the rationale as described by Ghattas et al. [76] is that the particular patient care pathways define a “context”, which can be related to a diagnosis or preferred course of action. Table 12 below tabulates some studies utilising predictive modelling.

**Table 12:** Examples of publications undertaking predictive modelling

References	Notable for
Jensen et al. [77]	disease trajectories reconstructed from free text in the electronic health records, used to quantify risk of subsequent clinical events adjusted for confounding factors
Benevento et al. [78]	Machine learning predicting waiting time from parameters derived from de facto pathways
Zhang and Padman [79]	Prediction of disease progression in multimorbid patients with 75% accuracy
Huang et al. [80, 81] Chen et al. [82]	Treatment pattern models trained for clinical outcome prediction using Topic Mining of derived
Li et al. [83]	Bayesian modelling approach to prediction of readmission

##### Resource analysis

Typically, resource analysis in this context is concerned with quantifying patient pathways according to their demand on services, whether directly through medical care or measured by proxy through key performance indicators (KPIs) such as cost or waiting time. Table 13 summarises some examples of approaches focussing primarily on cost, while Table 14 considers some examples focussing on resource utilisation and service redesign.

**Table 13:** Examples of publications undertaking resource analysis from a cost perspective

References	Notable for
Garg et al. [84]	Derivation of a Markov-type model of care pathways with associated costs from a long-term longitudinal database
Dahlin and Reharjo [85]	Statistical significance measures used to determine that implementation of a defined care pathway did not universally reduce costs in a multi-site study
Stefanini et al. [86]	Application of Time-Derived Activity Based Costing, validated against separate dataset
Zhang and Padman [87]	Assessment of variability of medication cost in multimorbidity using similarity determination of derived care pathways

**Table 14:** Examples of publications focussing on resource utilisation and service redesign

Reference	Notable for
Ceglowski et al. [88] Durojaiye et al. [89] Rojas et al. [90, 91] Abo-Hamad [92]	Analyses of resource allocation in emergency departments. Derived pathways examined with regard to assigned triage levels to consider appropriateness of assigned triage [89]; using patient disposition [90, 91] or medical roles and locations [92] with methods from [93] to identify bottlenecks in care; or using clustering methods to determine notably invariant temporal patterns in procedures performed [88]. All publications make recommendations for service reconfiguration or changes to practice
Stefanini et al. [94] Canjels et al. [95] Yoo et al. [96]	Focus on the use of process mining analyses to support implementation of a new unit [94]; recommend expansion of a satellite facility [95]; and assess effects of an implemented change of location [96]

##### Optimisation and simulation

In this context, both optimisation and simulation techniques are concerned with the modifications needed to a process model to achieve improvement in some KPI(s), whether the modifications are carried out manually or by an optimisation protocol.

Seven of the surveyed publications that utilise optimisation or simulation deal with implementations of queueing theory on models constructed using derived care pathways, while a further five use different optimisation techniques with reference to the physical layout of healthcare facilities.

A different approach to simulating the behaviour of a derived or modified process model is discrete event simulation (DES), where individual agents possessing attributes representative of the cohort as a whole progress through the states of the derived model with probabilities ascertained from the source data. The advantage is that both the attributes of the agents (patients) and the model are amenable to change. Six authors in the literature surveyed present implementations of discrete event simulation derived from patient care process models, though the topic is more frequently referred to in the literature.

Tables 15, 16 and 17 below consider some examples from the literature surveyed.

**Table 15:** Examples of publications utilising queueing theory for simulation and/or optimisation of care processes

References	Notable for
Yampaka et al. [97]	Transitions between states in a data-derived process model modelled as queues, allowing the effects of changes to staffing or patient numbers to be determined
Halonen et al. [98]	Comprehensive full life-cycle multi-method approach to data-driven service reconfiguration. Cycles of redesign and optimisation of resource allocation in a queueing network model informed experimental pilot studies to assess realistic working practices
Senderovich et al. [99]	Fork/join queueing network derived from administrative logs and schedules and Real Time Location Service (RTLS) data of an outpatient service allows simulation of different central pharmacy service policies. The optimal strategy is modelled to yield a 20% increase in performance

**Table 16:** Examples of publications applying discrete event simulation

References	Notable for
Zhou et al. [100] Kovalchuk et al. [101]	DES of derived care pathways focussing on different models of resource allocation to optimise patient waiting times
Augusto et al. [102]	Preliminary DES model assessing cost-effectiveness
Johnson et al. [11]	Portfolio of three case studies using models from a fully developed process mining framework (ClearPath method) to implement the NETIMIS health economics discrete event simulation tool [103], illustrating both the difficulties and the potential of this type of application. One fully successful case study is considered a regional exemplar of data driven care pathway improvement; in another, the process mining fails but successful simulation using an expert consensus model provides costed pathway improvements; and in the third case, failure of process mining to identify a clearly defined pathways identifies an urgent need for service improvement, presented to the relevant professional association

**Table 17:** Examples of publications undertaking simulation and/or optimisation with reference to physical layout

References	Notable for
Gartner et al. [104] Arnolds and Gartner [105] Rismanchian and Lee [106]	Optimisation of physical layouts based on derived de facto pathways
Meng et al. [107]	Assessment of changing patient numbers on functional area utilisation
Schwartz et al. [108]	Optimisation of scheduling with regard to bed and staff allocation incorporating various practical constraints

##### Statistical modelling

Several publications utilise statistical methods to implement supplementary techniques, as for example in the previously described publications of Nuemi et al. [73] and Li et al. [83], implementing clustering and predictive modelling respectively. Descriptive statistics are also commonplace, particularly where resource analysis or conformance analysis is applied. Our definition of statistical modelling as a supplemental technique in its own right is described in Table 7, capturing those publications where the results of statistical analysis are the main output aside from the process model. Relatively few publications can be so classified, less than half of the next most uncommon technique. It may be the case that statistical methods alone tend to serve to develop further methodologies rather than being an end in themselves; certainly, some of the authors below have published quite widely in this field using other techniques. Some examples of studies applying statistical modelling are tabulated in Table 18 below.

**Table 18:** Examples of publications classified as undertaking statistical modelling

References	Notable for
Liu et al. [109] Huang et al. [21]	Statistical analysis of associations within a symptom-diagnosis-treatment model [109]; and between derived pathways and treatments using probabilistic topic models [21]
Ibanez-Sanchez et al. [110] Fernandez-Llatas et al. [111]	Statistical analysis of admission times for different groups of patient pathways, extended to show significant effect of departmental reorganisation
Vogt et al. [112]	Outcome analysis including odds of hospitalisation for a very large and disparate set of pathways
Findlay et al. [113]	Extensive analysis of varied care pathways and outcomes populating a pre-defined pathway model
Yu et al. [114]	“Care Pathway Workbench”, facilitating guideline and statistical outcome analysis of patient pathways

#### Care pathways derived from other perspectives

Models where the activities of a care process are of an administrative/clinical nature comprise the substantial majority of the literature surveyed (85%). This likely relates to our initial search term; clinical care pathways and the various synonyms and related terms tend to have at least some administrative context, as opposed to clinical protocols or practice guidelines where the context in which care is delivered is often left unspecified. We also excluded a number of publications where an association is data mined from electronic records but no patient treatment paths are constructed. Of the alternative process models found, we shall briefly consider derived pathways from the role interaction and physical position perspectives here, and the perspective of clinical context in the “Clinical context perspective” section below. Table 19 presents some examples of role interaction models, while Table 20 summarises some literature using RTLS data to provide a physical position perspective.

**Table 19:** Examples of publications considering pathways from a role interaction perspective

References	Notable for
Alvarez et al. [115]	Some resource analysis on simple but informative models of staff role interactions differentiated according to patient triage level and diagnosis
Krutanard et al. [116] Huo et al. [117] Miranda et al. [118] Conca et al. [119]	Hierarchical clustering [116] and social network modelling [117–119]. Filtering from the departmental perspective allows insights on strategic departments and seasonal variation [118], while associations are found between biomarkers, patterns of collaboration, and outcomes in [119]

**Table 20:** Examples of publications considering pathways from a physical position perspective

References	Notable for
Fernandez-Llatas et al. [120]	Visualisation suite allowing filtering of process maps based on physical location
Kato-Lin and Padman [121]	Optimisation of patient waiting time, applying a constrained Markov Reward Process to derived model of sequences of transitions between care workers
Araghi et al. [122, 123] Miclo et al. [124]	Analysis of process efficiency and capacity using care sequence model from RTLS data; ascertains only 15% of patient time spent waiting

#### Clinical context perspective

Clinical context process models, where the sequence of events or activities described in the process model are of disease or treatment, are relatively uncommon in the literature surveyed, comprising just over 10% of the total. We believe this is as a consequence of the exclusion of publications where an association is data mined, but patient care processes are not reconstructed. Some examples of similar methodologies where care pathways are at least partially derived are presented in Table 21; Table A2 in Additional file 1: Appendix A classifies the 26 publications of this type according to supplementary technique and enhancing data.

**Table 21:** Examples of publications considering pathways from a clinical context perspective

References	Notable for
Williams et al. [125] Weber et al. [126] Boytcheva et al. [127] Dauxais et al. [128] Guyet et al. [129]	Clinical process models utilising prescription data, focussing on therapeutic decisions [125], polypharmacy [126], chronic comorbidities [127], and drug interactions in chronic disease [128, 129]. [126] identifies potential strong drug interactions in nearly 40% of patients, while [127] finds a statistical association between a particular initial treatment and a subsequent comorbidity. [128, 129] identify a particular change of medication associated with a subsequent acute episode in previously stable patients
Dabek et al. [130]	Visualisation tool allowing exploration of treatment pathways and comorbidities of a very large patient cohort
Blum et al. [132] Neumuth et al. [133, 134]	Clinical process models deriving workflows from transcribed video. [131] assesses utility of a checklist in improving guideline conformance, while [132–134] derive consensus surgical workflows. These are linked to video in [132], and are editable and mergeable in [133, 134]
Rojas and Capurro [135] Chen et al. [136] Movahedi et al. [137]	Patterns of treatment [135, 136] or adverse events [137]. [137] further determines clinically meaningful Markov Chain models of grouped adverse events
Riaño et al. [138]	State-Decision-Action model, where clinical practice is mined from treatment records to construct a data-derived clinical algorithm

## Discussion

The results of the systematic search above indicate the ongoing interest in derivation of patient de facto care pathways from electronic records. This has been facilitated by the ongoing development of frameworks for process mining in healthcare; in their exposition of the ClearPath method for generation of models suitable for simulation, Johnson et al. [11] identify four previous frameworks, methodologies or models by which process mining in healthcare should proceed. Gatta et al. [139] also present the Ste and pMineR packages as tools in a PM4HC (Process Mining for Healthcare) framework.

A general trend towards practical application of care pathway derivation methods can be discerned, with an increasing number of authors framing their analysis in terms of a particular research question or in the context of service redesign. A number of more recent papers follow Garg et al. [84] in using metrics of resource usage or cost as enhancing information [11, 86, 87, 95, 140, 141]; combined with the continuing interest in simulations modelled from derived care pathways described in the “Optimisation and simulation” section, this comprehensive use of data in resource planning and service redesign should find increasing application in health systems under continual pressure to maximise efficiency. In the broader context, clinical pathway redesign is increasingly data-facilitated if not always data-driven; a good example is the recent well publicised report of Connell et al. [142], where DeepMind (a subsidiary of Alphabet Inc.) essentially generated a portable implementation of a real-time updated electronic care record to facilitate a streamlined Acute Kidney Injury care pathway. Unfortunately their published evaluation did not analyse derived care pathways before and after implementation, rather simply comparing aggregate outcomes from the old and new pathways.

With regard to conceptual assessments of the utility of process mining within healthcare, the recent publications of Dahlin et al. [140] and Johnson [143] are of interest, taking an overview of the operation of healthcare systems and considering the place of process mining within them. Johnson places process mining as applied to healthcare in the framework of emergent complexity described by General Systems Theory (GST); a holistic and pragmatic approach is emphasised, as “*the only real certainty is that data will be different between systems and over time*”. The challenge is illustrated by the apposite comment that current medical devices are regulated on the basis of being rule based systems, but current developments both in medical AI and system complexity go well beyond those capabilities: GST is proposed to have utility in helping the adjustment to these technologies. Certainly, any theoretical model or framework which could aid the analysis of the decisions made within the complex social and administrative network of healthcare is welcome, particularly in the analysis of data-derived care pathways. Garcia et al. [144] performed a comparison of an EHR-based logistic regression model of intensive care management referral with thematic analysis of the decisions of the practitioners involved, finding that while their model had good (*c* = 0.75) predictive ability “*there remain “electronically unmeasured” factors that are important contributors to defining good referral candidates*”. The existence of such factors must be taken into account if data-driven care pathways are to play a role in formal care pathway redesign.

Dahlin et al. also advocates a pragmatic approach to the application of process mining in healthcare informed by current healthcare management practices. Process mining is discussed as a complement to the “process mapping” that is a key component of the discipline of health system Quality Improvement (QI) [145], enabling variation both by location and over time to be captured. The protocol of Litchfield et al. [146] is interesting to note in this context, proposing to explicitly contrast process mining and process mapping of practice at four UK primary care practices. Dahlin et al. reference the 2014 review of Yang and Su to show how limited applications of process mining in QI have been, an assertion partially borne out by the literature search presented above. Certainly, many publications have developed techniques that could readily be used be used to enhance QI, and a number comment on how results have been applied in practice. Nevertheless very few authors develop their work within a formal QI context and we agree with the proposal of Dahlin et al. that “*empirical research is needed about how process mining can be integrated into quality improvement of patient pathways and healthcare processes*”.

Finally, the discussions above may be granted greater relevance by the recent COVID-19 pandemic. The capacity of health systems to reconfigure themselves rapidly and effectively has been clearly demonstrated in very many instances around the world. Accurate and timely knowledge of the de facto pathways experienced by patients and the insights that can be gained by application of the analytical techniques surveyed in this review might have permitted more precise management of the responses to management of non-COVID19 care undertaken in many hospital and primary care services.

## Conclusion

In this study, we evaluated four review questions concerning the context in which care pathway derivation has been implemented in healthcare systems worldwide, and the potential of technology to aid in formal care pathway redesign. A limitation of the approach taken is that the topic surveyed is very broad, limiting discussion of methodological issues. On the other hand, we believe that our review provides an indication of the variety of ways in which methods utilising data-driven determination of de facto patient care pathways can provide relevant empirical information to those responsible for healthcare planning, management, and clinical practice. It is clear from this survey that despite the numbers of publications found the topic reviewed is as yet in its infancy, and we look forward to reports from those projects currently being implemented in healthcare practice.

## Supplementary Information

**Additional file 1.** **Appendix A:** Full classification of literature with clinical domain. **Table A1:** Publications deriving an administrative/clinical process model, classified by supplemental technique and enhancing data. **Table A2:** Publications deriving a clinical process model, classified by supplemental technique and enhancing data. **Appendix B:** Further discussion of selected literature.

## Data Availability

All data generated or analysed during this study are included in this published article and its Additional file 1.

## Abbreviations

ACS: Acute Coronary Syndrome
CHF: Congestive Heart Failure
CKD: Chronic Kidney Disease
DES: Discrete Event Simulation
EBM: Evidence Based Medicine
ED: Emergency Department
FCA: Formal Concept Analysis
GST: General Systems Theory
Hb: Haemoglobin
HMM: Hidden Markov Model
KPI: Key Performance Indicator
NICE: National Institute for Health and Care Excellence
NLP: Natural Language Processing
NSAID: Nonsteroidal Anti-Inflammatory Drug
PSA: Prostate-Specific Antigen
QI: Quality Improvement
RTLS: Real-Time Location Service
