# Supplementary material for: Clinical and operational insights from data-driven care pathway mapping: a systematic review
Source: BMC Med Inform Decis Mak. 2022 Feb 17;22:43. doi: 10.1186/s12911-022-01756-2 (PMC8851723; doi:10.1186/s12911-022-01756-2)
Supplement: Supplementary file 1 — Additional file 1. Appendix A: Full classification of literature with clinical domain. Table A1: Publications deriving an administrative/clinical process model, classified by supplemental technique and enhancing data. Table A2: Publications deriving a clinical process model, classified by supplemental technique and enhancing data. Appendix B: Further discussion of selected literature. [file 12911_2022_1756_MOESM1_ESM.docx]

# Appendix A: Full classification of literature with clinical domain

Table A1: Publications deriving an administrative/clinical process model, classified by supplemental technique and enhancing data

|  | No supp. technique | Clustering | Visualisation | Statistical Modelling | Predictive Modelling | Resource Analysis | Conformance Analysis | Simulation / Optimisation |
| --- | --- | --- | --- | --- | --- | --- | --- | --- |
| No enhancing data | Chen et al (2017)(1): A&E | Chen et al (2018)(2): inpatient processes | Glicksberg et al (2019)(3): gastroenterology | Huang et al (2014b)(4): oncology | Dart et al (2003)(5): inpatient processes | Ceglowski et al (2005)(6): A&E | Ainsworth & Buchan (2012)(7): Nephrology | Cho et al (2014)(8): outpatient processes |
|  | Dogan (2018)(9): outpatient processes | Elghazel et al (2007)(10): general medicine |  |  | Huang et al (2016)(11): cardiology | Chen et al (2015)(12): inpatient processes | Badakhshan & Alibabaei (2018)(13): ambulance processes | Gartner et al (2015)(14): inpatient processes |
|  | Fei et al (2010)(15): Surgery | Hirano & Tsumoto (2013a, 2013b)(16, 17): surgery | Happe & Drezen (2018)(18): General medicine |  | Huang, Z; Juarez, J; et al (2013)(19): respiratory medicine | Erdogan & Tarhan (2018)(20): surgery | Caron et al (2014a)(21): Oncology | Zhou,Z. et al (2014)(22): outpatient processes |
|  | Fox et al (2018)(23): dentistry | Kirchner et al (2016)(24): surgery | Kumar et al (2014)(25): respiratory medicine |  | Kopec et al (2004)(26): intensive care | Garg et al (2009)(27): geriatrics | Caron et al (2013)(28): Oncology |  |
|  | Ganesha et al (2017a, 2017b)(29, 30): inpatient processes | Montani & Leonardi (2014)(31): neurology | Trutt, Mauduit & Leclère (2019)(32): surgery |  | Le Duff et al (2001)(33): general practice | Helbig et al (2015)(34): inpatient processes | Caron et al (2011)(35): Oncology |  |
|  | Garg & Agarwal (2016)(36): inpatient processes | Naeem et al (2017)(37): gastroenterology |  |  | Lin et al (2001)(38): neurology | Lismont et al (2016)(39): endocrinology | De Vries et al (2017)(40): A&E |  |
|  | Huang et al (2013a)(41): oncology | Prokofyeva, Zaytsev & Maltseva(2019)(42): general medicine |  |  | Lin et al (2002)(43): nephrology | Mans, Schonenberg, Song, Van Der Aalst, Bakker (2008)(44): oncology | Gatta et al (2017)(45): oncology |  |
|  | Huang et al (2013c)(46): neurology | Tsumoto et al (2012a, 2012b, 2012c, 2013a, 2015a, 2015b, 2015c, 2016)(47-54): nursing |  |  | Lin et al (2005)(55): obstetrics | Mans et al (2012)(56): dentistry | Haq et al (2016)(57): Public health |  |
|  | Iwata et al (2012, 2013b)(58, 59): nursing | Tsumoto et al (2018)(60): neurology |  |  | Li et al (2019)(61): cardiology | Perimal-Lewis et al (2012)(62): inpatient processes | Huang et al (2012c)(63): cardiology |  |
|  | Kurniati et al (2018)(64): oncology |  |  |  |  | Rebuge & Ferreira (2012)(65): A&E | Huang et al (2014a)(66): oncology |  |
|  | Lang et al (2008)(67): diagnostic imaging |  |  |  |  | Riz et al (2016)(68): oncology | Huang et al (2015c)(69): cardiology |  |
|  | Le et al (2019)(70, 71): urology |  |  |  |  | Stefanini et al (2017)(72): oncology | Tsumoto et al (2013b, 2013c)(73, 74): nursing |  |
|  | Ozkaynak & Brennan (2013)(75): A&E |  |  |  |  | Tsumoto et al (2014)(76): nursing | Van de Klundert et al (2010)(77): cardiology |  |
|  | Perimal-Lewis et al (2014)(78): inpatient processes |  |  |  |  |  | Yoo et al (2015)(79, 80): surgery |  |
|  | Prodel et al (2015,2018)(80, 81): cardiology |  |  |  |  |  |  |  |
|  | Senderovich, Weidlich & Gal (2019)(82): oncology |  |  |  |  |  |  |  |
|  | Stefanini et al (2016)(83): oncology |  |  |  |  |  |  |  |
|  | Williams et al (2014)(84): neurology |  |  |  |  |  |  |  |
|  | Zhang & Chen (2012)(85): outpatient processes |  |  |  |  |  |  |  |
| Outcomes | Baker et al (2017)(86): Oncology | Lakshmanan et al (2013)(87): cardiology | Perer & Gotz (2013)(88): cardiology | Findlay et al (2018)(89): cardiology |  |  | *Lenkowicz et al(2018)(90): oncology* | Augusto et al (2017)(91): Cardiology |
|  | Li et al (2015)(92): endocrinology/cardiology | Montani et al (2014)(93): neurology | Najjar et al (2018)(94): multimorbidity | Vogt et al (2017)(95): outpatient processes |  |  | Li,X.;Mei,J.;Liu,H.;Yu,Y.;Xie,G.;Hu,J.;Wang,F (2015)(96): cardiology | Kovalchuk et al (2018)(97): cardiology |
|  |  | *Ozkaynak et al (2015)*(98)*: respiratory medicine* | Yamashita et al (2015)(99): surgery |  |  |  | Poelmans et al (2010)(100): oncology | Xia et al (2019)(101):endocrinology/cardiology |
| Biomarkers | Kaymak et al (2012)(102): anaesthetics | Dagliati et al (2014)(103): Endocrinology | Bettencourt-Silva et al (2015)(104): Oncology |  | *Funkner et al (2017b)*(105)*: cardiology* |  |  |  |
|  |  | Dagliati et al (2017)(106): Oncology | Bettencourt-Silva et al (2016)(107): Oncology |  |  |  |  |  |
|  |  | Dagliati et al (2018)(108): Endocrinology | Basole (2014)(109): Resp. medicine |  |  |  |  |  |
|  |  | *Funkner et al (2017b)*(105)*: cardiology* |  |  |  |  |  |  |
| Guidelines | Xu,X.;Jin,T.;Wang,J.(2016)(110): neurology | Villamil et al (2017)(111): oncology | *Caballero et al (2017)(112): general medicine* | Yu et al (2014)(113): cardiology | Ghattas et al (2010)(114): infectious disease | Mannhardt & Blinde (2017)(115): A&E | Antonelli et al (2012)(116): Oncology | Yeo (2017)(117): orthopedics |
|  | Xu et al (2017b)(118): neurology/surgery |  | Fernandez-Llatas et al (2015)(119): Endocrinology |  |  |  | Binder et al (2012)(120): Oncology |  |
|  | Williams et al (2019)(121): multimorbidity |  | Fernandez-Llatas et al (2016)(122): Cardiology |  |  |  | Bohada et al (2012)(123): Cardiology |  |
|  |  |  |  |  |  |  | *Caballero et al (2017)(112): general medicine* |  |
|  |  |  |  |  |  |  | Huang et al (2012b)(124): oncology |  |
|  |  |  |  |  |  |  | Iwata et al (2013a, 2013c)(125, 126): nursing |  |
|  |  |  |  |  |  |  | Kamišalić et al (2018)(127): cardiology |  |
|  |  |  |  |  |  |  | Li, X et al (2014)(128): cardiology |  |
|  |  |  |  |  |  |  | Molodchenkov & Khachumov (2016)(129): Resp. medicine |  |
|  |  |  |  |  |  |  | Rovani et al (2015)(130): urology |  |
|  |  |  |  |  |  |  | Xu,X.;Jin,T.;Wei,Z.;Lv,C.;Wang,J. (2016)(131): neurology |  |
| Comorbidities / Complications |  | Maruster & Jorna (2005)(132): vascular surgery | Huang, C-W et al (2015)(133): nephrology | Liu, L et al (2013)(134): endocrinology | Huang et al (2016) (135): cardiology |  |  |  |
|  |  | Yan,C. et al (2016)(136): cardiology | *Zhang,Y.;Padman,R.;Patel,N. (2015)*(137)*: nephrology* |  | Jensen et al (2017)(138): oncology |  |  |  |
|  |  | Zhang et al (2014)(139): nephrology | *Zhang & Padman (2017)*(140)*: nephrology/endocrinology* |  | Liu, R (2015)(141): cardiology |  |  |  |
|  |  | *Zhang,Y.;Padman,R.;Wasserman,L.;Patel,N.;Teredesai,P.;Xie,Q. (2015)*(142)*: nephrology* |  |  | Zhang & Padman (2015)(143): nephrology |  |  |  |
| Prescriptions |  | *Zhang,Y.;Padman,R.;Wasserman,L.;Patel,N.;Teredesai,P.;Xie,Q. (2015)*(142)*: nephrology* | Perer et al (2015)(144): cardiology |  |  |  | Han et al (2011)(145): Endocrinology |  |
|  |  |  | *Zhang,Y.;Padman,R.;Patel,N. (2015)*(137)*: nephrology* |  |  |  |  |  |
|  |  |  | *Zhang & Padman (2017)*(140)*: nephrology/endocrinology* |  |  |  |  |  |
| Clinical Classification | Bose & Van Der Aalst (2011)(146): Oncology | Delias et al (2015)(147): A&E | Basole (2015)(148): Resp. medicine | *Ibanez-Sanchez et al (2019)(149): neurology* | Huang, Z et al (2015a)(150): cardiology | Abo-Hamad (2017)(151): A&E |  | Lamine et al (2015)(152): ambulance processes |
|  |  |  |  |  |  | Durojaiye et al (2018)(153): A&E |  |  |
|  |  |  |  |  |  | Neira et al (2019)(154): A&E |  |  |
|  | Defossez et al (2014)(155): Oncology | Lee et al (2011)(156): endocrinology | *Ozkaynak et al (2015)* (98)*: respiratory medicine* |  |  | Rojas et al (2019a, 2019b)(157, 158): A&E |  | *Rismanchian & Lee (2017)*(159)*: A&E* |
|  | Rojas et al (2017)(160): A&E |  |  |  |  | Zhang & Padman (2016)(161): nephrology/endocrinology |  |  |
| Physical Information | Helmering et al (2012)(162): Cardiology | Hilton et al (2016)(163): respiratory medicine | Hirano & Tsumoto (2014, 2015)(164, 165): outpatient processes |  | Egho et al (2013)(166): Oncology | *Dahlin & Raharjo (2019)(167): oncology* |  | Arnolds & Gartner (2017)(168): Operational |
|  | Mans, Schonenberg, Leonardi, Panzarasa, Cavallini, Quaglini, Van Der Aalst (2008)(169): Neurology | *Ozkaynak et al (2015)* (98)*: respiratory medicine* |  |  |  | Yoo et al (2016)(170): outpatient processes |  | Halonen et al (2017)(171): dentistry |
|  | Partington et al (2015)(172): Cardiology | Suriadi et al (2014)(173): cardiology |  |  |  |  |  | Meng et al (2016)(174): A&E |
|  |  | Nuemi et al (2013)(175): oncology |  |  |  |  |  | *Rismanchian & Lee (2017) )*(159)*: A&E* |
|  |  |  |  |  |  |  |  | Schwarz, Römer & Mellouli (2019)(176): urology |
|  |  |  |  |  |  |  |  | Senderovich, Rogge-Solti et al (2015)(177): oncology |
|  |  |  |  |  |  |  |  | Senderovich, Weidlich et al (2015)(178): oncology |
|  |  |  |  |  |  |  |  | Senderovich et al (2016)(179): oncology |
|  |  |  |  |  |  |  |  | Vahdat et al (2019)(180): outpatient processes |
| Other medical data | Andrews et al (2019a, 2019b)(181, 182): ambulance services | Funkner et al (2017a)(183): cardiology | Klimov et al (2010)(184): oncology | Fernandez-Llatas et al (2018)(185): neurology | Benevento et al (2019)(186): A&E | Canjels et al (2019)(187): orthopedics | Cho et al (2020)(188): surgery | Johnson et al (2018)(189): General medicine/neurology |
|  | Gicquel et al (2015)(190): oncology | Shen et al (2012)(191): oncology |  | Huang et al(2018)(192): cardiology | Li, C et al (2016)(193): cardiology | *Dahlin & Raharjo (2019)(167): oncology* | Dewandono et al (2013)(194): Endocrinology | Mans, Reijers, Wismeijer, van Genuchten (2013)(195): dentistry |
|  | Liu, H et al (2015)(196): cardiology |  |  | *Ibanez-Sanchez et al (2019)(149): neurology* | Meier et al (2015)(197):oncology | Kim et al (2013)(198): outpatient processes | Huang, Z; Gan, C et al (2013)(199): oncology | Yampaka & Chongstitvatana (2016)(200): outpatient processes |
|  | Wang et al (2017)(201): endocrinology/cardiology/neurology |  |  |  | Mohammed & Benlamri (2014)(202): cardiology | Stefanini et al (2019)(203): oncology | Huang et al (2012a)(204): endocrinology |  |
|  | Uragaki et al (2016)(205): urology |  |  |  | Yang, S.; Li, J.; et al (2017)(206): A&E |  | Huang et al (2013b)(207): oncology |  |
|  | Xu et al (2017a)(208): neurology/surgery |  |  |  |  |  | Hwang et al (2004)(209): obs & gynae |  |
|  | Yang, S.; Zhou, M.; et al (2017)(210): A&E |  |  |  |  |  | Kirchner et al (2013)(211): surgery |  |
|  |  |  |  |  |  |  | *Lenkowicz et al(2018)(90): oncology* |  |
|  |  |  |  |  |  |  | Mans, van der Aalst, Vanwersch (2013)(212): oncology |  |
|  |  |  |  |  |  |  | Wang et al (2014)(213): surgery |  |
|  |  |  |  |  |  |  | Yan, H. et al (2017)(214): cardiology |  |
|  |  |  |  |  |  |  | Yang & Hwang (2006)(215): obs & gynae |  |
|  |  |  |  |  |  |  | Zhou, M. et al (2017)(216): A&E |  |

Publications in italics have substantial use of more than one supplementary technique or enhancing data type.

Table A2: Publications deriving a clinical process model, classified by supplemental technique and enhancing data

|  | No supp. Technique | Visualisation | Statistical Modelling | Predictive Modelling | Resource Analysis | Conformance Analysis | Simulation / Optimisation |
| --- | --- | --- | --- | --- | --- | --- | --- |
| No enhancing data | Guyet et al (2017)(217): pharmacology | Blum et al (2008)(218): surgery | Adeyemi et al (2013)(219): resp. medicine | Du et al (2012)(220): oncology |  |  | Du et al (2013)(221): obs. & gynae. |
|  | Movahedi et al (2019)(222): cardiology | Dabek et al (2015)(223): neurology | Chen et al (2015)(224): inpatient processes | Huang et al (2015)(225): cardiology |  |  |  |
|  | Riaño et al (2008)(226): resp. medicine |  | Neumuth et al (2011)(227): surgery |  |  |  |  |
|  | Rojas & Capurro (2019)(228): intensive care |  |  |  |  |  |  |
|  | Williams et al (2017)(229): cardiology |  |  |  |  |  |  |
| Outcomes |  |  |  | Chen et al (2019)(230): General medicine | Chen et al (2018)(231): neurology |  |  |
| Guidelines |  |  | Kelleher et al (2014)(232): A&E |  |  |  |  |
| Comorbidities / Complications | Dauxais et al (2017)(233): pharmacology |  |  |  |  |  |  |
|  | Sun et al (2013)(234): endocrinology |  |  |  |  |  |  |
| Prescriptions | Boytcheva et al (2016)(235): multimorbidity |  |  |  |  |  |  |
| Physical Information | Tóth et al (2017) (236): oncology |  |  |  |  |  |  |
| Other medical data | McGregor (2011)(237): intensive medicine |  |  | Shknevsky et al (2017)(238): endocrinology |  | Huang et al (2014)(239): cardiology | Neumuth et al (2012)(240): surgery |
|  | Valero-Ramon et al (2019)(241): geriatrics |  |  |  |  |  |  |
|  | Weber et al (2018)(242): multimorbidity |  |  |  |  |  |  |

# Appendix B: Further discussion of selected literature

*Additional information for section 3.4.1.1: Publications not utilising supplemental techniques*

A good example of a focus on methodology can be found in the 2014 publication of Williams et al (84); the patient journeys of 1078 patients with suspected stroke are reconstructed from the Salford Integrated Record using string matching of events transcribed as symbols, and particular methodologies are shown to be superior to an alternative when the data used has been recorded in the incorrect sequence. Another example of a methodological focus is how Le et al (71) demonstrate a method for adding noise to records for analysis to enhance privacy. The 2018 publication of Prodel et al (80) illustrates all three motivations: they develop and verify a novel method, and claim optimal information content and improved computational efficiency in the reconstruction of patient pathways from recorded data. Their study on 1602 patients with implanted cardiac resynchronisation defibrillators derives complication, readmission, and mortality data from the process model, and is intended to be translated into formalisms suitable for direct use in simulation models.

Publications which utilise enhancing data, but which we do not consider to apply supplemental techniques, tend to be of two types. In the first type, the derived care pathways are compared against the enhancing data, or the enhancing data partitions the process models. For example, Uragaki et al (205) compared the sequencing of a consensus clinical pathway for cryptorchidism fusion surgery derived by physicians of the University of Miyazaki Hospital with a typical care pathway extracted from the hospital EHR system, finding close correspondence for treatments involving medication, but substantial variation for non-pharmacological interventions. Extracting a different dataset from the source previously described, Williams et al (121) analyse prescribing practice and adverse events in UK primary care with regard to guidelines on the use of Nonsteroidal Anti-Inflammatory Drugs (NSAIDs). An example of different process models being derived according to physical position is the 2008 study of Mans et al (169), utilising the GLADIS clinical database to carry out a comparison of derived care pathways for ischemic stroke patients at four hospitals in the Italian region of Lombardy and uncover differences in the prevalence of neuroprotection and antihypertensive therapy. Similarly, Partington et al (172) compare the care pathways of chest pain admissions at four South Australian hospitals, confirming existing stakeholder assumptions of variation in clinical practice.

In the second approach, the enhancing data is incorporated into the process model. An example is the 2017 publication of Baker et al (86), where a comprehensive Markov model is developed from clinical records to describe the derived care pathways of 955 patients undergoing multiple cycles of two different chemotherapy regimens along with their outcomes, developing a detailed picture of the frequency and context of complications such as neutropenia. The developed model is explicitly intended to be similar to those used in the field of health economics, facilitating future quantitative research in health technology assessment.

*Additional information for section 3.4.1.2: Conformance Analysis*

A good example of the potential of conformance analysis to improve clinical care can be found in the 2010 publication of Poelmans et al (100). In this work, the derived care pathways (including clinical indicators) of 148 patients being treated for Primary Operable Breast Cancer at a Belgian hospital were modelled using Hidden Markov Models (HMM) and Formal Concept Analysis (FCA). A subset of patients with a length of stay >=10 days was identified who experienced a substantially more complex care pathway due to clinical complications and additional morbidities. The FCA lattice method also allowed identification of a previously overlooked quality of care issue, where insufficient pain medication was being administered before the removal of wound drains; and a patient specific issue where failure to consult history might have resulted in inappropriate nursing care. Extension of this approach to consider process variations among different types of breast cancer surgery identified a counter-intuitively greater complexity of the care process for the less technically complex breast conserving surgery as opposed to mastectomy, arising from significant missing care interventions. It was determined that reductions in time spent as an inpatient rendered the prescribed care pathway impossible to execute, and that a redesign was required.

Another approach to combining conformance analysis of derived care pathways with outcome data is the 2015 publication of Li et al (96). Here, a Chinese care pathway for congestive heart failure (CHF) is captured as 22 constraints describing the temporal dependencies and contraindications of multiple pharmacological therapies, which is used to create a set of care pathway variation patterns that are applicable to real-world data. 8193 CHF patients are separated into positive and negative outcome groups based on subsequent hospitalisation, and the odds-ratios for the effect of a variation in practice on the outcome was determined.

*Additional information for section 3.4.1.4: Predictive modelling*

Notably, Jensen et al (138) derive care pathways using disease trajectories reconstructed from free text in the electronic health records of a Norwegian hospital to quantify the risk of subsequent clinical events for cancer patients, adjusted for confounding factors. Benevento et al (186) apply machine learning to parameters derived from patient progress through emergency department de facto pathways to predict waiting time. Zhang and Padman (143) use a different approach to predict with up to 75% accuracy the disease progression of multimorbid patients with chronic kidney disease.

Predictive modelling has also been undertaken to attempt to predict clinical outcomes from a patients derived care pathway, through the training of a treatment pattern model using topic mining (11, 19, 230). Li et al (193) utilise a Bayesian modelling approach to predict 14 day readmission for patients with polyvascular disease.

*Additional information for section 3.4.1.5: Resource Analysis*

Examining first examples of publications where cost is the focus, Garg et al (27) utilised a large longitudinal database of geriatric admissions at St Georges Hospital, London, to derive a type of Markov model of care process pathways with associated costs. The publication is notable for the exceptionally long timescale considered, derived as it is from 18 years of registry data. Also examining costs, Dahlin & Reharjo (167) is notable for its multisite approach comparing breast cancer treatment at four Swedish hospitals; the use of statistical significance measures; and the finding that implementing a defined care pathway did not necessarily reduce costs at all sites. Stefanini et al (203) apply Time-Derived Activity Based Costing to their derived lung cancer treatment process models, finding good agreement between their derived resource usage and a validation dataset provided by the hospital.

Zhang & Padman (161) used similarity determination of derived care pathways to classify multimorbid patients (CKD stage 3, diabetes, hypertension) into three subgroups by clinical complexity. Group statistics on the prevalence of disease progression and patient medication costs were calculated. The same statistics were then derived for patients assigned to subgroup according to the quartile of their medication costs alone. A correspondence was found between measures of the mean clinical complexity and medication cost, but with a very high variability in cost: illustrative of this, the highest medication cost found was for a patient classified as being of medium clinical complexity.

Ceglowski et al (6), Durojaiye et al (153), Rojas et al (157, 158), and Abo-Hamad (151) consider the care pathways experienced by users of emergency departments; Ceglowski et al in a major Australian metropolitan hospital, Durojaiye et al in a level 1 US paediatric trauma centre, Rojas et al in an academic hospital in Santiago (Chile), and Abo-Hamad at a large Dublin (Ireland) A&E department with significant capacity issues.

Ceglowski et al derived clusters of de facto care pathways from one year of records, discovering that the mix of procedures performed by the emergency department did not vary on an hourly, daily, weekly and seasonal basis. They utilised this result to propose resource planning of shift changes to simply take account of variation in the number of presentations to the ED. The publications of Rojas et al and Abo-Hamad analyse derived care pathways with regard to assigned triage levels using the “fuzzy miner” of Gunther and Van der Aalst, and identify particular bottlenecks slowing patients appropriate treatment. Durojaiye et al also consider assigned triage levels, but use a different algorithm and focus on the appropriateness of triage level assignment. Rojas et al focus on patient disposition at discharge and diagnosis, while Abo-Hamad quantifies the medical roles providing care processes and the locations at which they are provided. Abo-Hamad further focusses on service reconfiguration: Previous manually derived process models used for simulation are held to have yielded effective improvement strategies, but the author considers that the latency caused by updating the process model manually hampers timely decision making. The author indicates that a project is underway to integrate the process mining engine with the Hospital Information System to allow real-time tracking of emergency department processes to facilitate informed resource allocation.

Stefanini et al (72) and Canjels et al (187) concern themselves with resource analysis to support service redesign. Stefanini et al describe how process mapping has been used to support the implementation of a patient focussed care lung cancer unit in the University Hospital of Pisa, based on 470 cases from 2014. The process model is refined with the aid of medical experts, and the average demand per patient on various services quantified, with the aim of predicting the resources and facilities required for the new unit. Service redesign is also the focus of Canjels et al, where analysis of the derived care pathways supports expansion of an outlier clinic of Maastricht University Medical Centre for treatment of less complex knee osteoarthritis patients.

Finally, the assessment of an already reconfigured service is the aim of Yoo et al (170). They derived process models for outpatient process at the cancer and clinical neuroscience centres of Seoul National Bundang Hospital, for one month of 2012 and 2013. In the interim the centres moved to a new building; the main KPI assessed was waiting times, which did not increase despite a substantial increase in patient numbers.

*Additional information for section 3.4.1.6: Simulation and optimisation*

These methods can be implemented in various ways. For example, Yampaka et al (200) derive timing information from a process model based on 334 outpatient records of a Thai hospital, allowing them to model transitions between states in the process model as queues. They then determine the effect of adding staff or increasing patient numbers on both patient waiting time and cost.

A comprehensive description of service reconfiguration using a multimethod approach, including simulation techniques supplemental to a data-derived process model, is found in Halonen et al (171). The authors conducted process improvement at a Finnish dental clinic, which wished to modify its service model to minimise missed appointments by consolidating initial and return appointments into one visit. This work is notable for covering the full lifecycle of service reconfiguration, from initial scoping, through data collection and model building to pilot and then full implementation. Resource allocation in a queueing network model was optimised according to cost and patient throughput KPIs, and cycles of redesign and optimisation informed experimental pilot studies to assess realistic working practices.

Senderovich et al (179) combine conformance analysis and performance analysis through simulation, using datasets relating to outpatient chemotherapy treatment visits to the Dana Farber Cancer Institute in Boston. By combining data from pharmacy event logs, patient locations recorded by a Real Time Location Service (RTLS), and the appointment schedule they derive a fork/join queueing network, capable of modelling scheduled services and accounting for delays arising from both waiting for resources and waiting for concurrent processes to finish. The data indicates that a number of patients experience delays in their visit arising from waiting for medication to be prepared by the central pharmacy, while nurses are available to administer it. Simulations of the use of different service policies for the pharmacy (for example, first-come first-served or shortest processing first) indicate that changing the service policy to first-come first-served would yield a 20% increase in performance.

A different approach to simulating the behaviour of a derived or modified process model is discrete event simulation (DES), where individual agents possessing attributes representative of the cohort as a whole progress through the states of the derived model with probabilities ascertained from the source data. The advantage is that both the attributes of the agents (patients) and the model are amenable to change. Six authors in the literature surveyed present implementations of discrete event simulation derived from patient care process models, though the topic is more frequently referred to in the literature.

Zhou et al (22) use records from a Chicago outpatient clinic to derive a process model, which is then simplified by utilising another process mining algorithm, yielding a model suitable for conversion to a DES simulation. They find good convergence between simulated performance indexes and the results of analysis of the records, and identify the bottleneck in the workflow. They use the simulation model to determine that reassigning a receptionist role to a nursing role would substantially disimprove the patient waiting time; and the upper bound for the number of doctors deployed, beyond which patient waiting time is not substantially reduced. A similar focus on optimal provision of services is found in Kovalchuk et al’s analysis of the flow of Acute Coronary Syndrome (ACS) patients at a St Petersburg cardiology centre (97). A discrete event simulation is designed where simulated patients are assigned characteristics of one of the derived care pathways; the resulting simulation is explored with varying patient flow rates and facility availabilities, to determine the effect on waiting time for angiography, an important clinical parameter for ACS patients.

Augusto et al (91) present a preliminary DES model derived from process mining French national hospitalisation data for patients with implantable cardioverter defibrillators, taking a cost-effectiveness analysis approach to compare different implantation and device replacement strategies. The authors comment that the model is not sufficiently complex to capture all the mechanisms at work, but it is to be hoped that the novel process mining framework recently described by Prodel et al (80) will facilitate more successful simulation efforts.

A fully developed process mining framework intended for use in the NETIMIS health economics discrete event simulation tool is described by Johnson et al as the ClearPath method (189). They present three UK case studies of simulation models informing hospital practice, which illustrate both the difficulties and the potential of this type of application. In the case study on alcohol-related emergency admissions, electronic care records were successfully process mined to develop a DES model, which is considered a regional exemplar of data driven care pathway improvement. Attempts at process mining electronic health records for giant cell arteritis are unsuccessful, and the authors relied on interviews with clinical experts to derive their DES model, which provides a costed model for pathway improvement. The final case study considers the derived care pathways of patients presenting with functional neurological symptoms. Process mining of audit data derives a very complex “spaghetti” process model, not suitable for deriving a simulation from; however, the details of this undefined pathway illustrate an urgent need for service improvement, presented to the relevant professional association.

Finally, we consider those publications which concern themselves with optimisation of the physical layout of healthcare facilities. Arnolds and Gartner, in two publications (14, 168), derive the sequence of locations followed by patient care pathways at a 350 bed German hospital. They then use a mathematical model for hospital layout planning to determine the optimal allocation of specialties to the hospital floorplan, taking account of the derived care pathways. Rismanchian & Lee (159) use a conceptually similar approach to determine a layout of the emergency department of a Korean hospital which would reduce the distances travelled by critical and non-critical patients by 42% and 47% respectively.

Taking a slightly different emphasis, Meng et al (174) derive the de facto care pathways of patients of the emergency department of a Singaporean hospital, utilising a mathematical model to determine the patient flow across different areas of the emergency department. By classifying the patients by diagnosis-related group, they then assess the impact of changes in patient numbers on the utilisation of different functional areas. The issue of scheduling to optimise bed and staff allocation for a urology department with respect to particular constraints (e.g. single-gender rooms) is the focus of Schwarz et al (176), who use a hierarchical mixed integer linear programming approach based on data-derived care pathways.

*Additional information for section 3.4.1.7: Statistical Modelling*

Liu et al (134) correlate diseases, complications and treatments for a cohort of 177,000 geriatric diabetes patients. Using different methods, Huang et al (4) analyse the relationship between discovered patterns in derived care pathways and treatments from a statistical point of view. Ibanez-Sanchez et al (149) and Fernandez-Llatas et al (185) analyse the treatment of acute stroke in an emergency department, showing statistically significant differences in admission time for different groups of patients. They extend their analysis to show a statistically significant effect from a recent re-organisation of the department.

Vogt, Scholz & Sundmacher (95) present an analysis of derived care pathways and outcomes for German heart failure outpatients from a large health insurance dataset covering approximately one third of the population. The context of their study emphasises how important an understanding of the operation of a healthcare system is to the performance of care pathway derivation: their analysis takes into account that in the German healthcare system there is no “gatekeeper” either medically or administratively, so an outpatient may begin their care pathway with either a general practitioner or a specialist, and may attend different practitioners simultaneously. One aspect of their analysis thus focuses on statistical analysis of the odds of hospitalisation according to the sequence of physician specialisms attended.

Findlay et al (89) present an analysis using data linkage to build an e-registry deriving care pathways and outcomes of Scottish ACS patients. They are somewhat of an outlier in this review, as they populated a pre-defined process model rather than using a data-driven approach to process model definition; it could be argued that this should exclude them from this review. However, they do present an extensive analysis in terms of varied care pathways and outcomes.

Finally, Yu et al (113) describe a Care Pathway Workbench, where sequences of clinical events extracted from historical practice data are combined with guidelines and statistical outcome analysis.

*Additional information for section 3.4.3: Clinical Context Perspective*

The Salford Integrated Record was used by Williams et al (229) to build a clinical process model of therapeutic decisions from extracted prescriptions for 81,096 hypertensive patients over a 37 year period. Prescriptions in UK primary care are likewise the focus of Weber et al (242), focussing on the appropriateness of polypharmacy for six common chronic conditions. They determine potential strong drug interactions for nearly 40% of patients in their dataset. Again utilising prescription data to enhance the model, on a larger scale Boytcheva et al (235) mined the Bulgarian National Health Insurance fund for chronic comorbidities of schizophrenia, diabetes and hyperprolactinemia, presenting derived pathways from a disease perspective and finding a statistical association between initial treatment for schizophrenia and subsequent type 2 diabetes.

Likewise within the domain of pharmacoepidemiology Dauxais et al (233) and Guyet et al (217) utilised the French SNIIRAM database to extract prescriptions for patients with stable antiepileptic treatment to determine a speculative drug interaction, and identified a particular change of medication which preceded seizures in 20 previously stable patients. Considering other techniques, a visualisation tool is developed by Dabek et al (223), allowing the exploration of the treatment paths and comorbidities of a very large cohort of patients subsequent to a first traumatic brain injury.

A second theme in publications deriving clinical process models is the evaluation of clinical workflows, often determined from transcribed video. With the exception of Kelleher et al (232), who assessed whether a checklist improved conformance with guidelines in paediatric trauma resuscitation, this use of annotated video footage is restricted to surgical workflow modelling, as performed by Blum et al (218), and Neumuth et al (227, 240). In these cases a consensus surgical workflow is built up which describes the process of surgery; Blum et al link the consensus workflow to video of the surgery, while Neumuth et al develop a surgical workflow editor, and a methodology for merging different workflows.

Patterns of treatment are determined by for example Rojas & Capurro (228), from the MIMIC-II intensive care database; or by Chen et al (231), from three traditional Chinese medicine hospitals treating cerebral infarct. Rather than treatments, Movahedi et al (222) derive sequences of adverse events post implantation of a Left Ventricular Assist Device, from which they determine clinically meaningful Markov Chain models of grouped adverse events.

Finally, Riaño et al (226) develop a state-decision-action model, where clinical practice is mined from treatment records to construct a data-derived clinical algorithm.

References

1. Chen S, Yang S, Zhou M, Burd RS, Marsic I. Process-oriented Iterative Multiple Alignment for Medical Process Mining. arXiv preprint arXiv:1709.05440. 2017.

2. Chen Y, Kho AN, Liebovitz D, Ivory C, Osmundson S, Bian J, et al. Learning bundled care opportunities from electronic medical records. Journal of Biomedical Informatics. 2018;77:1-10.

3. Glicksberg BS, Oskotsky B, Thangaraj PM, Giangreco N, Badgeley MA, Johnson KW, et al. PatientExploreR: an extensible application for dynamic visualization of patient clinical history from electronic health records in the OMOP common data model. Bioinformatics. 2019;35(21):4515-8.

4. Huang Z, Dong W, Ji L, Gan C, Lu X, Duan H. Discovery of clinical pathway patterns from event logs using probabilistic topic models. J Biomed Informatics. 2014;47:39-57.

5. Dart T, Cui Y, Chatellier G, Degoulet P. Analysis of hospitalised patient flows using data-mining. Studies in Health Technology and Informatics; 18th International Congress of the European Federation for Medical Informatics, MIE 2003; 4 May 2003 through 7 May 2003; St. Malo. ; 2003.

6. Ceglowski A, Churilov L, Wassertheil J. Knowledge discovery through mining emergency department data. Proceedings of the Annual Hawaii International Conference on System Sciences; 38th Annual Hawaii International Conference on System Sciences; 3 January 2005 through 6 January 2005; Big Island, HI. ; 2005.

7. Ainsworth J, Buchan I. COCPIT: A tool for Integrated Care Pathway Variance Analysis. Studies in Health Technology and Informatics; 24th Medical Informatics in Europe Conference, MIE 2012; 26 August 2012 through 29 August 2012; Pisa. ; 2012.

8. Cho M, Song M, Yoo S. A systematic methodology for outpatient process analysis based on process mining. Brisbane, QLD edSpringer Verlag; 2014 [cited 13 March 2018].

9. Dogan O. Process mining for check-up process analysis. IIOABJ. 2018;9(6):56-61.

10. Elghazel H, Deslandres V, Kallel K, Dussauchoy A. Clinical pathway analysis using graph-based approach and Markov models. 2007 2nd International Conference on Digital Information Management, ICDIM; 2007 2nd International Conference on Digital Information Management, ICDIM; 28 October 2007 through 31 October 2007; Lyon. ; 2007.

11. Huang Z, Dong W, Ji L, Duan H. Predictive monitoring of clinical pathways. Expert Sys Appl. 2016;56:227-41.

12. Chen Y, Xie W, Gunter CA, Liebovitz D, Mehrotra S, Zhang H, et al. Inferring Clinical Workflow Efficiency via Electronic Medical Record Utilization. AMIA Annu Symp Proc. 2015;2015:416-25.

13. Badakhshan P, Alibabaei A. Using process mining for process analysis improvement in pre-hospital emergency. Middle East North Africa Conference for Information Systems, Paris; ; 2018.

14. Gartner D, Arnolds IV, Nickel S. Improving Hospital-wide Patient Scheduling Decisions by Clinical Pathway MiningIOS Press; 2015 [cited 20 February 2018].

15. Fei H, Meskens N. Discovering patient care process models from event logs. Conférence Internationale de Modélisation et Simulation (MOSIM 10), Special track on Healthcare Information systems and decision making; ; 2010.

16. Hirano S, Tsumoto S. Mining clinical pathway candidates from order history based on the clustering of order sequences. Proceedings - 2013 IEEE International Conference on Systems, Man, and Cybernetics, SMC 2013; 2013 IEEE International Conference on Systems, Man, and Cybernetics, SMC 2013; 13 October 2013 through 16 October 2013; Manchester. ; 2013.

17. Hirano S, Tsumoto S. Clustering of order sequences based on the typicalness index for finding clinical pathway candidates. Proceedings - IEEE 13th International Conference on Data Mining Workshops, ICDMW 2013; 2013 13th IEEE International Conference on Data Mining Workshops, ICDMW 2013; 7 December 2013 through 10 December 2013; Dallas, TX. IEEE Computer Society; 2013.

18. Happe A, Drezen E. A visual approach of care pathways from the French nationwide SNDS database – from population to individual records: the ePEPS toolbox. Fundam Clin Pharmacol. 2018;32(1):81-4.

19. Huang Z, Juarez JM, Duan H, Li H. Length of stay prediction for clinical treatment process using temporal similarity. Expert Sys Appl. 2013;40(16):6330-9.

20. Gurgen Erdogan T, Tarhan A. A Goal-Driven Evaluation Method Based On Process Mining for Healthcare Processes. Applied Sciences. 2018;8(6).

21. Caron F, Vanthienen J, Vanhaecht K, Limbergen EV, De Weerdt J, Baesens B. Monitoring care processes in the gynecologic oncology department. Comput Biol Med. 2014;44(1):88-96.

22. Zhou Z, Wang Y, Li L. Process mining based modeling and analysis of workflows in clinical care - A case study in a chicago outpatient clinic. Proceedings of the 11th IEEE International Conference on Networking, Sensing and Control, ICNSC 2014; 11th IEEE International Conference on Networking, Sensing and Control, ICNSC 2014; 7 April 2014 through 9 April 2014; Miami, FL. IEEE Computer Society; 2014.

23. Fox F, Aggarwal VR, Whelton H, Johnson O. A Data Quality Framework for Process Mining of Electronic Health Record Data. ; 2018.

24. Kirchner K, Marković P, Delias P. AUTOMATIC CREATION OF CLINICAL PATHWAYS–A CASE STUDY. DATA SCIENCE AND BUSINESS INTELLIGENCE 179. 2016:188.

25. Kumar V, Park H, Basole RC, Braunstein M, Kahng M, Chau DH, et al. Exploring clinical care processes using visual and data analytics: challenges and opportunities. Proceedings of the 20th ACM SIGKDD conference on knowledge discovery and data mining workshop on data science for social good; ; 2014.

26. Kopec D, Shagas G, Reinharth D, Tamang S. Development of a clinical pathways analysis system with adaptive Bayesian Nets and data mining techniques. Studies in Health Technology and Informatics; International Congress on Medical and Care Compunetics, ICCMC 2004; 2 June 2004 through 4 June 2004; The Hague. ; 2004.

27. Garg L, McClean S, Meenan B, Millard P. Non-homogeneous Markov models for sequential pattern mining of healthcare data. IMA J Manage Math. 2009;20(4):327-44.

28. Caron F, Vanthienen J, Baesens B. Healthcare Analytics: Examining the Diagnosis–treatment Cycle. Procedia Technology. 2013;9:996-1004.

29. Ganesha K, Supriya KV, Soundarya M. Analyzing the waiting time of patients in hospital by applying heuristics process miner. Proceedings of the International Conference on Inventive Communication and Computational Technologies, ICICCT 2017; 2017 International Conference on Inventive Communication and Computational Technologies, ICICCT 2017; 10 March 2017 through 11 March 2017; Institute of Electrical and Electronics Engineers Inc.; 2017.

30. Ganesha K, Soundarya M, Supriya KV. The best fit process model for the utilization of the physical resources in hospitals by applying inductive visual miner. Proceedings of the International Conference on Inventive Communication and Computational Technologies, ICICCT 2017; 2017 International Conference on Inventive Communication and Computational Technologies, ICICCT 2017; 10 March 2017 through 11 March 2017; Institute of Electrical and Electronics Engineers Inc.; 2017.

31. Montani S, Leonardi G. Non-exhaustive trace retrieval for managing stroke patients; 2014 [cited 13 March 2018].

32. Trutt L, Mauduit N, Leclère B. Development of a Graphical Interface to Visualize and Analyze the Pathways of Patients During Their Hospital Stay for Thoracic Surgery. Stud Health Technol Inform. 2019;264:1882-3.

33. Le Duff F, Happe A, Burgun A, Levionnois S, Bremond M, Le Beux P. Sharing medical data for patient path analysis with data mining method. Medinfo. 2001;10(Pt 2):1364-8.

34. Helbig K, Römer M, Mellouli T. A clinical pathway mining approach to enable scheduling of hospital relocations and treatment servicesSpringer Verlag; 2015 [cited 20 February 2018].

35. Caron F, Vanthienen J, De Weerdt J, Baesens B, De Weerdt J, Baesens B. Beyond x-raying a care-flow: Adopting different focuses on care-flow mining. Proceedings of the First International Business Process Intelligence Challenge (BPIC11). 2011:1-11.

36. Garg N, Agarwal S. Process mining for clinical workflows. ACM International Conference Proceeding Series; 2016 International Conference on Advances in Information Communication Technology and Computing, AICTC 2016; 12 August 2016 through 13 August 2016; Association for Computing Machinery; 2016.

37. Naeem MR, Ali W, Hamad Naeem MA, Abro WA. A multi-level process mining framework for correlating and clustering of biomedical activities using event logs. International Journal of Advanced Computer Science and Applications. 2017;8(3):393-401.

38. Lin F-, Chou S-, Pan S-, Chen Y-. Mining time dependency patterns in clinical pathways. Int J Med Inform. 2001;62(1):11-25.

39. Lismont J, Janssens A-, Odnoletkova I, vanden Broucke S, Caron F, Vanthienen J. A guide for the application of analytics on healthcare processes: A dynamic view on patient pathways. Comput Biol Med. 2016;77:125-34.

40. de Vries G, Neira RAQ, Geleijnse G, Dixit P, Mazza BF. Towards Process Mining of EMR Data. BIOSTEC 2017. 2017:585.

41. Huang Z, Lu X, Duan H. Similarity measuring between patient traces for clinical pathway analysis. Murcia edSpringer Verlag; 2013 [cited 20 February 2018].

42. Prokofyeva ES, Zaytsev RD, Maltseva SV. Application of Modern Data Analysis Methods to Cluster the Clinical Pathways in Urban Medical Facilities. ; 2019.

43. Lin F-, Chiu C-, Wu S-. Using Bayesian networks for discovering temporal-state transition patterns in Hemodialysis. Proceedings of the Annual Hawaii International Conference on System Sciences; 35th Annual Hawaii International Conference on System Sciences, HICSS 2002; 7 January 2002 through 10 January 2002; IEEE Computer Society; 2002.

44. Mans RS, Schonenberg MH, Song M, Van Der Aalst, W. M. P., Bakker PJM. Application of process mining in healthcare - A case study in a Dutch Hospital. Funchal, Madeira ed; 2008 [cited 15 March 2018].

45. Gatta R, Vallati M, Lenkowicz J, Rojas E, Damiani A, Sacchi L, et al. Generating and comparing knowledge graphs of medical processes using pMineR. Proceedings of the Knowledge Capture Conference, K-CAP 2017; 9th International Conference on Knowledge Capture, K-CAP 2017; 4 December 2017 through 6 December 2017; Association for Computing Machinery, Inc; 2017.

46. Huang Z, Lu X, Duan H. Latent treatment topic discovery for clinical pathways. J Med Syst. 2013;37:1-10.

47. Tsumoto S, Hirano S, Iwata H. Temporal data mining for nursing schedule management. Proceedings - 3rd International Conference on Innovations in Bio-Inspired Computing and Applications, IBICA 2012; 3rd International Conference on Innovations in Bio-Inspired Computing and Applications, IBICA 2012; 26 September 2012 through 28 September 2012; Kaohsiung City. ; 2012.

48. Tsumoto S, Hirano S, Iwata H. Data-oriented maintenance of clinical pathway using clustering and multidimensional scaling. Conference Proceedings - IEEE International Conference on Systems, Man and Cybernetics; 2012 IEEE International Conference on Systems, Man, and Cybernetics, SMC 2012; 14 October 2012 through 17 October 2012; Seoul. ; 2012.

49. Tsumoto S, Iwata H, Hirano S. Construction andmaintenance of clinical pathways using data mining methods. International Conference on Information and Knowledge Management, Proceedings; 2nd International Workshop on Managing Interoperability and Complexity in Health Systems, MIX-HS 2012, Collocated with the 21st ACM International Conference on Information and Knowledge Management, CIKM 2012; 29 October 2012 through 29 October 2012; Maui, HI. ; 2012.

50. Tsumoto S, Hirano S, Iwata H. Mining clinical pathway using clustering and rule induction. Proceedings - 2013 IEEE International Conference on Systems, Man, and Cybernetics, SMC 2013; 2013 IEEE International Conference on Systems, Man, and Cybernetics, SMC 2013; 13 October 2013 through 16 October 2013; Manchester. ; 2013.

51. Tsumoto S, Hirano S, Iwata H. Mining schedule of nursing care based on dual-clustering. ; 2015.

52. Tsumoto S, Hirano S, Iwata H. Data decomposition and dual clustering for clinical care management. Proceedings - 2015 IEEE International Conference on Big Data, IEEE Big Data 2015; 3rd IEEE International Conference on Big Data, IEEE Big Data 2015; 29 October 2015 through 1 November 2015; Institute of Electrical and Electronics Engineers Inc.; 2015.

53. Tsumoto S, Hirano S, Iwata H. Dual clustering and data decomposition for nursing care management. ACM International Conference Proceeding Series; ASE BigData and SocialInformatics, ASE BD and SI 2015; 7 October 2015 through 9 October 2015; Association for Computing Machinery; 2015.

54. Tsumoto S, Hirano S, Iwata H. Construction of clinical pathway from histories of clinical actions in hospital information system. Proceedings - 2016 IEEE International Conference on Big Data, Big Data 2016; 4th IEEE International Conference on Big Data, Big Data 2016; 5 December 2016 through 8 December 2016; Institute of Electrical and Electronics Engineers Inc.; 2016.

55. Lin F-, Hsieh L-, Pan S-. Learning clinical pathway patterns by hidden markov model. Proceedings of the Annual Hawaii International Conference on System Sciences; 38th Annual Hawaii International Conference on System Sciences; 3 January 2005 through 6 January 2005; Big Island, HI. ; 2005.

56. Mans RS, Reijers HA, Van Genuchten M, Wismeijer D. Mining processes in dentistry. IHI'12 - Proceedings of the 2nd ACM SIGHIT International Health Informatics Symposium; 2nd ACM SIGHIT International Health Informatics Symposium, IHI'12; 28 January 2012 through 30 January 2012; Miami, FL. ; 2012.

57. Haq R, Pires SCV, Kapp JM, Schlemper S, Simoes EJ. Using Process Mining to Assess the Fidelity of a Home Visiting Program. Frontiers in Public Health Services and Systems Research. 2016;5(4):5-11.

58. Iwata H, Tsumoto S, Hirano S. Data-oriented construction and maintenance of clinical pathway using similarity-based data mining methods. Proceedings - 12th IEEE International Conference on Data Mining Workshops, ICDMW 2012; 12th IEEE International Conference on Data Mining Workshops, ICDMW 2012; 10 December 2012 through 10 December 2012; Brussels. ; 2012.

59. Iwata H, Tsumoto S, Hirano S. Clinical schedule management based on granularity-based mining. 2013 IEEE International Conference on Cybernetics, CYBCONF 2013; 2013 IEEE International Conference on Cybernetics, CYBCONF 2013; 13 June 2013 through 15 June 2013; Lausanne. ; 2013.

60. Tsumoto S, Hirano S, Kimura T, Iwata H. Construction of Clinical Pathway Generation from Nursing Records and Discharge Summaries. ; 2018.

61. Li J, Tan X, Xu X, Wang F. Efficient Mining Template of Predictive Temporal Clinical Event Patterns From Patient Electronic Medical Records. IEEE Journal of Biomedical and Health Informatics. 2019;23(5):2138-47.

62. Perimal-Lewis L, Qin S, Thompson C, Hakendorf P. Gaining insight from patient journey data using a process-oriented analysis approach. Proceedings of the Fifth Australasian Workshop on Health Informatics and Knowledge Management-Volume 129; Australian Computer Society, Inc.; 2012.

63. Huang Z, Lu X, Duan H. Anomaly detection in clinical processes. AMIA Annu Symp Proc. 2012;2012:370-9.

64. Kurniati AP, Hall G, Hogg D, Johnson O. Process mining in oncology using the MIMIC-III dataset. Journal of Physics: Conference Series. 2018;971:012008.

65. Rebuge A, Ferreira DR. Business process analysis in healthcare environments: A methodology based on process mining. Inf Syst. 2012;37(2):99-116.

66. Huang Z, Lu X, Duan H. Similarity measuring between patient traces for clinical pathway analysis. Murcia edSpringer Verlag; 2013 [cited 15 March 2018].

67. Lang M, Bürkle T, Laumann S, Prokosch H-. Process mining for clinical workflows: Challenges and current limitations. Studies in Health Technology and Informatics; ; 2008.

68. Riz G, Santos EAP, Loures, Eduardo De Freitas Rocha. Process Mining to Knowledge Discovery in Healthcare Processes. ISPE TE; ; 2016.

69. Huang Z, Dong W, Ji L, Yin L, Duan H. On local anomaly detection and analysis for clinical pathways. Artif Intell Med. 2015;65(3):167-77.

70. Le HH, Edman H, Honda Y, Kushima M, Yamazaki T, Araki K, et al. Fast Generation of Clinical Pathways including Time Intervals in Sequential Pattern Mining on Electronic Medical Record Systems. ; 2017.

71. Le HH, Kushima M, Araki K, Yokota H. Differentially private sequential pattern mining considering time interval for electronic medical record systems. Proceedings of the 23rd International Database Applications & Engineering Symposium; ; 2019.

72. Stefanini A, Aloini D, Dulmin R, Mininno V. Service reconfiguration in healthcare systems: The case of a new focused hospital unit. Springer Proceedings in Mathematics and Statistics; 3rd International Conference on Health Care Systems Engineering, HCSE 2017; 29 May 2017 through 31 May 2017; Springer New York LLC; 2017.

73. Tsumoto S, Hirano S, Iwata H. Granularity-based mining for construction of nursing care plan. Proceedings of the 12th IEEE International Conference on Cognitive Informatics and Cognitive Computing, ICCI*CC 2013; 12th IEEE International Conference on Cognitive Informatics and Cognitive Computing, ICCI*CC 2013; 16 July 2013 through 18 July 2013; New York, NY. ; 2013.

74. Tsumoto S, Hirano S, Iwata H. Mining nursing care plan from data extracted from hospital information system. Proceedings of the 2013 IEEE/ACM International Conference on Advances in Social Networks Analysis and Mining, ASONAM 2013; 2013 IEEE/ACM International Conference on Advances in Social Networks Analysis and Mining, ASONAM 2013; 25 August 2013 through 28 August 2013; Niagara Falls, ON. Association for Computing Machinery; 2013.

75. Ozkaynak M, Brennan P. An observation tool for studying patient-oriented workflow in hospital emergency departments. Methods Inf Med. 2013;52(6):503-13.

76. Tsumoto S, Iwata H, Hirano S, Tsumoto Y. Similarity-based behavior and process mining of medical practices. Future Gener Comput Syst. 2014;33:21-31.

77. van de Klundert J, Gorissen P, Zeemering S. Measuring clinical pathway adherence. Journal of Biomedical Informatics. 2010;43(6):861-72.

78. Perimal-Lewis L, De Vries D, Thompson CH. Health intelligence: Discovering the process model using process mining by constructing Start-to-End patient journeys. Proceedings of the Seventh Australasian Workshop on Health Informatics and Knowledge Management-Volume 153; Australian Computer Society, Inc.; 2014.

79. Prodel M, Augusto V, Xie X, Jouaneton B, Lamarsalle L. Discovery of patient pathways from a national hospital database using process mining and integer linear programming. IEEE International Conference on Automation Science and Engineering; 11th IEEE International Conference on Automation Science and Engineering, CASE 2015; 24 August 2015 through 28 August 2015; IEEE Computer Society; 2015.

80. Prodel M, Augusto V, Jouaneton B, Lamarsalle L, Xie X. Optimal Process Mining for Large and Complex Event Logs. IEEE Trans Autom Sci Eng. 2018.

81. Prodel M, Augusto V, Xie X, Jouaneton B, Lamarsalle L. Discovery of patient pathways from a national hospital database using process mining and integer linear programming. IEEE International Conference on Automation Science and Engineering; 11th IEEE International Conference on Automation Science and Engineering, CASE 2015; 24 August 2015 through 28 August 2015; IEEE Computer Society; 2015.

82. Senderovich A, Weidlich M, Gal A. Context-aware temporal network representation of event logs: Model and methods for process performance analysis. Information Systems. 2019;84:240-54.

83. Stefanini A, Aloini D, Dulmin R, Mininno V. Linking Diagnostic-Related Groups (DRGs) to their processes by process mining. HEALTHINF 2016 - 9th International Conference on Health Informatics, Proceedings; Part of 9th International Joint Conference on Biomedical Engineering Systems and Technologies, BIOSTEC 2016; 9th International Conference on Health Informatics, HEALTHINF 2016 - Part of 9th International Joint Conference on Biomedical Engineering Systems and Technologies, BIOSTEC 2016; 21 February 2016 through 23 February 2016; SciTePress; 2016.

84. Williams R, Buchan IE, Prosperi M, Ainsworth J. Using String Metrics to Identify Patient Journeys through Care Pathways. AMIA Annu Symp Proc. 2014;2014:1208-17.

85. Zhang X, Chen S. Pathway identification via process mining for patients with multiple conditions. IEEE International Conference on Industrial Engineering and Engineering Management; 2012 IEEE International Conference on Industrial Engineering and Engineering Management, IEEM 2012; 10 December 2012 through 13 December 2012; Hong Kong. IEEE Computer Society; 2012.

86. Baker K, Dunwoodie E, Jones RG, Newsham A, Johnson O, Price CP, et al. Process mining routinely collected electronic health records to define real-life clinical pathways during chemotherapy. Int J Med Informatics. 2017;103:32-41.

87. Lakshmanan GT, Rozsnyai S, Wang F. Investigating clinical care pathways correlated with outcomes. Beijing ed; 2013 [cited 10 January 2018].

88. Perer A, Gotz D. Data-Driven Exploration of Care Plans for Patients. Conference on Human Factors in Computing Systems - Proceedings; 31st Annual CHI Conference on Human Factors in Computing Systems:, CHI EA 2013; 27 April 2013 through 2 May 2013; Association for Computing Machinery; 2013.

89. Findlay I, Morris T, Zhang R, McCowan C, Shield S, Forbes B, et al. Linking hospital patient records for suspected or established acute coronary syndrome in a complex secondary care system: a proof-of-concept e-registry in National Health Service Scotland. Eur Heart J Qual Care Clin Outcomes. 2018;4(3):155-67.

90. Lenkowicz J, Gatta R, Masciocchi C, Casà C, Cellini F, Damiani A, et al. Assessing the conformity to clinical guidelines in oncology: An example for the multidisciplinary management of locally advanced colorectal cancer treatment. Management Decision. 2018;56(10):2172-86.

91. Augusto V, Xie X, Prodel M, Jouaneton B, Lamarsalle L. Evaluation of discovered clinical pathways using process mining and joint agent-based discrete-event simulation. Proceedings - Winter Simulation Conference; 2016 Winter Simulation Conference, WSC 2016; 11 December 2016 through 14 December 2016; Institute of Electrical and Electronics Engineers Inc.; 2017.

92. Li X, Liu H, Mei J, Yu Y, Xie G. Mining Temporal and Data Constraints Associated with Outcomes for Care Pathways. Stud Health Technol Inform. 2015;216:711-5.

93. Montani S, Leonardi G, Quaglini S, Cavallini A, Micieli G. Improving structural medical process comparison by exploiting domain knowledge and mined information. Artif Intell Med. 2014;62(1):33-45.

94. Najjar A, Reinharz D, Girouard C, Gagné C. A two-step approach for mining patient treatment pathways in administrative healthcare databases. Artificial Intelligence in Medicine. 2018;87:34-48.

95. Vogt V, Scholz SM, Sundmacher L. Applying sequence clustering techniques to explore practice-based ambulatory care pathways in insurance claims data. Eur J Public Health. 2017;28(2):214-9.

96. Li X, Mei J, Liu H, Yu Y, Xie G, Hu J, et al. Analysis of Care Pathway Variation Patterns in Patient Records. Studies in Health Technology and Informatics; 26th Medical Informatics in Europe Conference, MIE 2015; 27 May 2015 through 29 May 2015; IOS Press; 2015.

97. Kovalchuk SV, Funkner AA, Metsker OG, Yakovlev AN. Simulation of patient flow in multiple healthcare units using process and data mining techniques for model identification. Journal of Biomedical Informatics. 2018;82:128-42.

98. Ozkaynak M, Dziadkowiec O, Mistry R, Callahan T, He Z, Deakyne S, et al. Characterizing workflow for pediatric asthma patients in emergency departments using electronic health records. J Biomed Informatics. 2015;57:386-98.

99. Yamashita T, Flanagan B, Wakata Y, Hamai S, Nakashima Y, Iwamoto Y, et al. Visualization of key factor relation in clinical pathway. Procedia Computer Science; 19th International Conference on Knowledge Based and Intelligent Information and Engineering Systems, KES 2015; 7 September 2015 through 9 September 2015; Elsevier B.V.; 2015.

100. Poelmans J, Dedene G, Verheyden G, Van Der Mussele H, Viaene S, Peters E. Combining business process and data discovery techniques for analyzing and improving integrated care pathways. Berlin ed; 2010 [cited 15 January 2018].

101. Xia K, Zhong X, Zhang L, Wang J. Optimization of Diagnosis and Treatment of Chronic Diseases Based on Association Analysis Under the Background of Regional Integration. J Med Syst. 2019;43(3):46.

102. Kaymak U, Mans R, Van De Steeg T, Dierks M. On process mining in health care. Conference Proceedings - IEEE International Conference on Systems, Man and Cybernetics; 2012 IEEE International Conference on Systems, Man, and Cybernetics, SMC 2012; 14 October 2012 through 17 October 2012; Seoul. ; 2012.

103. Dagliati A, Sacchi L, Cerra C, Leporati P, De Cata P, Chiovato L, et al. Temporal data mining and process mining techniques to identify cardiovascular risk-associated clinical pathways in Type 2 diabetes patients. 2014 IEEE-EMBS International Conference on Biomedical and Health Informatics, BHI 2014; 2014 IEEE-EMBS International Conference on Biomedical and Health Informatics, BHI 2014; 1 June 2014 through 4 June 2014; Valencia. IEEE Computer Society; 2014.

104. Bettencourt-Silva JH, Clark J, Cooper CS, Mills R, Rayward-Smith VJ, De La Iglesia B. Building data-driven pathways from routinely collected hospital data: A case study on prostate cancer. JMIR Med.Informatics. 2015;3(3):0.

105. Funkner AA, Yakovlev AN, Kovalchuk SV. Data-driven modeling of clinical pathways using electronic health records. ; 2017.

106. Dagliati A, Sacchi L, Zambelli A, Tibollo V, Pavesi L, Holmes JH, et al. Temporal electronic phenotyping by mining careflows of breast cancer patients. J Biomed Informatics. 2017;66:136-47.

107. Bettencourt-Silva JH, Mannu GS, de la Iglesia B. Visualisation of integrated patient-centric data as pathways: Enhancing electronic medical records in clinical practice; 2016.

108. Dagliati A, Tibollo V, Cogni G, Chiovato L, Bellazzi R, Sacchi L. Careflow Mining Techniques to Explore Type 2 Diabetes Evolution. J Diabetes Sci Technol. 2018;12(2):251-9.

109. Basole RC, Park H, Kumar V, Braunstein ML, Bost J, Chau DH, et al. Bicentric visualization of pediatric asthma care process activities. Proceedings of IEEE VIS 2014 Workshop of Electronic Health Records, IEEE; ; 2014.

110. Xu X, Jin T, Wang J. Summarizing patient daily activities for clinical pathway mining. 2016 IEEE 18th International Conference on e-Health Networking, Applications and Services, Healthcom 2016; 18th IEEE International Conference on e-Health Networking, Applications and Services, Healthcom 2016; 14 September 2016 through 17 September 2016; Institute of Electrical and Electronics Engineers Inc.; 2016.

111. Villamil MDP, Barrera D, Velasco N, Bernal O, Fajardo E, Urango C, et al. Strategies for the quality assessment of the health care service providers in the treatment of Gastric Cancer in Colombia. BMC Health Serv Res. 2017;17(1).

112. Caballero HSG, Corvò A, Dixit PM, Westenberg MA. Visual analytics for evaluating clinical pathways. ; 2017.

113. Yu Y, Liu H, Li J, Li X, Mei J, Xie G, et al. Care Pathway Workbench: Evidence Harmonization from Guideline and DataIOS Press; 2014 [cited 15 March 2018].

114. Ghattas J, Peleg M, Soffer P, Denekamp Y. Learning the context of a clinical process. Ulm edSpringer Verlag; 2010 [cited 15 March 2018].

115. Mannhardt F, Blinde D. Analyzing the trajectories of patients with sepsis using process mining. CEUR Workshop Proceedings; Joint Radar Tracks at the 18th International Working Conference on Business Process Modeling, Development and Support, BPMDS 2017 and the 22nd International Working Conference on Evaluation and Modeling Methods for Systems Analysis and Development, EMMSAD 2017 and the 8th International Workshop on Enterprise Modeling and Information Systems Architectures, EMISA 2017; 12 June 2017 through 13 June 2017; CEUR-WS; 2017.

116. Antonelli D, Baralis E, Bruno G, Chiusano S, Mahoto NA, Petrigni C. Analysis of diagnostic pathways for colon cancer. Flexible Serv Manuf J. 2012;24(4):379-99.

117. Yeo HJ. Medical service improvement through patient’s queue decision mining. J Theor Appl Inf Technol. 2017;95(18):4853-61.

118. Xu X, Jin T, Wei Z, Wang J. Incorporating Topic Assignment Constraint and Topic Correlation Limitation into Clinical Goal Discovering for Clinical Pathway Mining. J Healthc Eng. 2017;2017.

119. Fernandez-Llatas C, Martinez-Millana A, Martinez-Romero A, Benedi JM, Traver V. Diabetes care related process modelling using Process Mining techniques. Lessons learned in the application of Interactive Pattern Recognition: Coping with the Spaghetti Effect. Proceedings of the Annual International Conference of the IEEE Engineering in Medicine and Biology Society, EMBS; 37th Annual International Conference of the IEEE Engineering in Medicine and Biology Society, EMBC 2015; 25 August 2015 through 29 August 2015; Institute of Electrical and Electronics Engineers Inc.; 2015.

120. Binder M, Dorda W, Duftschmid G, Dunkl R, Fröschl KA, Gall W, et al. On analyzing process compliance in skin cancer treatment: an experience report from the evidence-based medical compliance cluster (EBMC 2). International Conference on Advanced Information Systems Engineering; Springer; 2012.

121. Williams R, Ashcroft DM, Brown B, Rojas E, Peek N, Johnson O. Process Mining in Primary Care: Avoiding Adverse Events Due to Hazardous Prescribing. Stud Health Technol Inform. 2019;264:447-51.

122. Fernandez-Llatas C, Bayo JL, Martinez-Romero A, Benedi JM, Traver V. Interactive pattern recognition in cardiovascular disease management. A process mining approach. ; 2016.

123. Bohada JA, Riaño D, López-Vallverdú JA. Automatic generation of clinical algorithms within the state-decision-action model. Expert Systems with Applications. 2012;39(12):10709-21.

124. Huang Z, Lu X, Duan H. On mining clinical pathway patterns from medical behaviors. Artif Intell Med. 2012;56(1):35-50.

125. Iwata H, Tsumoto S, Hirano S. Maintenance of nursing care plan using similarity-based data mining methods. 2013 ICME International Conference on Complex Medical Engineering, CME 2013; 2013 7th ICME International Conference on Complex Medical Engineering, CME 2013; 25 May 2013 through 28 May 2013; Beijing. ; 2013.

126. Iwata H, Tsumoto S, Hirano S. Data mining based clinical care plan construction. 2013 International Joint Conference on Awareness Science and Technology and Ubi-Media Computing: Can We Realize Awareness via Ubi-Media?, iCAST 2013 and UMEDIA 2013; 2013 International Joint Conference on Awareness Science and Technology, iCAST 2013 and 6th International Conference on Ubi-Media Computing, UMEDIA 2013; 2 November 2013 through 4 November 2013; Aizuwakamatsu. IEEE Computer Society; 2013.

127. Kamišalić A, Riaño D, Welzer T. Formalization and acquisition of temporal knowledge for decision support in medical processes. Computer Methods and Programs in Biomedicine. 2018;158:207-28.

128. Li X, Liu H, Zhang S, Mei J, Xie G, Yu Y, et al. Automatic Variance Analysis of Multistage Care PathwaysIOS Press; 2014 [cited 16 January 2018].

129. Molodchenkov A, Khachumov M. Using the DTW method for estimation of deviation of care processes from a care plan. Аналитика и управление данными в областях с интенсивным использованием данных; ; 2016.

130. Rovani M, Maggi FM, De Leoni M, Van Der Aalst, W. M. P. Declarative process mining in healthcare. Expert Sys Appl. 2015;42(23):9236-51.

131. Xu X, Jin T, Wei Z, Lv C, Wang J. TCPM: Topic-Based Clinical Pathway Mining. Proceedings - 2016 IEEE 1st International Conference on Connected Health: Applications, Systems and Engineering Technologies, CHASE 2016; 1st IEEE International Conference on Connected Health: Applications, Systems and Engineering Technologies, CHASE 2016; 27 June 2016 through 29 June 2016; Institute of Electrical and Electronics Engineers Inc.; 2016.

132. Maruster L, Jorna RJ. From data to knowledge: a method for modeling hospital logistic processes. IEEE Transactions on Information Technology in Biomedicine. 2005;9(2):248-55.

133. Huang C, Lu R, Iqbal U, Lin S, Nguyen PA, Yang H, et al. A richly interactive exploratory data analysis and visualization tool using electronic medical records. BMC Medical Informatics and Decision Making. 2015;15(1):92.

134. Liu L, Tang J, Cheng Y, Agrawal A, Liao W-, Choudhary A. Mining diabetes complication and treatment patterns for clinical decision support. International Conference on Information and Knowledge Management, Proceedings; 22nd ACM International Conference on Information and Knowledge Management, CIKM 2013; 27 October 2013 through 1 November 2013; San Francisco, CA. ; 2013.

135. Huang Z, Dong W, Ji L, He C, Duan H. Incorporating comorbidities into latent treatment pattern mining for clinical pathways. J Biomed Informatics. 2016;59:227-39.

136. Yan C, Chen Y, Li B, Liebovitz D, Malin B. Learning Clinical Workflows to Identify Subgroups of Heart Failure Patients. AMIA Annu Symp Proc. 2016;2016:1248-57.

137. Zhang Y, Padman R, Patel N. Paving the COWpath: Learning and visualizing clinical pathways from electronic health record data. J Biomed Informatics. 2015;58:186-97.

138. Jensen K, Soguero-Ruiz C, Oyvind Mikalsen K, Lindsetmo R-, Kouskoumvekaki I, Girolami M, et al. Analysis of free text in electronic health records for identification of cancer patient trajectories. Sci Rep. 2017;7.

139. Zhang Y, Padman R, Wasserman L. On Learning and Visualizing Practice-based Clinical Pathways for Chronic Kidney Disease. AMIA Annu Symp Proc. 2014;2014:1980-9.

140. Zhang Y, Padman R. An interactive platform to visualize data-driven clinical pathways for the management of multiple chronic conditionsIOS Press; 2017 [cited 26 January 2018].

141. Liu R, Srinivasan RV, Zolfaghar K, Chin S-, Roy SB, Hasan A, et al. Pathway-finder: An interactive recommender system for supporting personalized care pathways. IEEE International Conference on Data Mining Workshops, ICDMW; 14th IEEE International Conference on Data Mining Workshops, ICDMW 2014; 14 December 2014; IEEE Computer Society; 2015.

142. Zhang Y, Padman R, Wasserman L, Patel N, Teredesai P, Xie Q. On clinical pathway discovery from electronic health record data. IEEE Intell Syst. 2015;30(1):70-5.

143. Zhang Y, Padman R. Innovations in chronic care delivery using data-driven clinical pathways. Am J Manag Care. 2015;21(12):e661-8.

144. Perer A, Wang F, Hu J. Mining and exploring care pathways from electronic medical records with visual analytics. J Biomed Informatics. 2015;56:369-78.

145. Han B, Jiang L, Cai H. Abnormal process instances identification method in healthcare environment. Proc. 10th IEEE Int. Conf. on Trust, Security and Privacy in Computing and Communications, TrustCom 2011, 8th IEEE Int. Conf. on Embedded Software and Systems, ICESS 2011, 6th Int. Conf. on FCST 2011; 10th IEEE Int. Conf. on Trust, Security and Privacy in Computing and Communications, TrustCom 2011, 8th IEEE Int. Conf. on Embedded Software and Systems, ICESS 2011, 6th Int. Conf. on Frontier of Computer Science and Technology, FCST 2011; 16 November 2011 through 18 November 2011; Changsha. ; 2011.

146. Bose RJC, van der Aalst, Wil MP. Analysis of Patient Treatment Procedures. Business Process Management Workshops (1); ; 2011.

147. Delias P, Doumpos M, Grigoroudis E, Manolitzas P, Matsatsinis N. Supporting healthcare management decisions via robust clustering of event logs. Knowl Based Syst. 2015;84:203-13.

148. Basole RC, Braunstein ML, Kumar V, Park H, Kahng M, Chau DH(, et al. Understanding variations in pediatric asthma care processes in the emergency department using visual analytics. J Am Med Inform Assoc. 2015;22(2):318.

149. Ibanez-Sanchez G, Fernandez-Llatas C, Martinez-Millana A, Celda A, Mandingorra J, Aparici-Tortajada L, et al. Toward Value-Based Healthcare through Interactive Process Mining in Emergency Rooms: The Stroke Case. International Journal of Environmental Research and Public Health. 2019;16(10).

150. Huang Z, Dong W, Bath P, Ji L, Duan H. On mining latent treatment patterns from electronic medical records. Data Min Knowl Discov. 2015;29(4):914-49.

151. Abo-Hamad W. Patient pathways discovery and analysis using process mining techniques: An emergency department case study. Springer Proceedings in Mathematics and Statistics; 3rd International Conference on Health Care Systems Engineering, HCSE 2017; 29 May 2017 through 31 May 2017; Springer New York LLC; 2017.

152. Lamine E, Fontanili F, Mascolo MD, Pingaud H. Improving the management of an emergency call service by combining process mining and discrete event simulation approachesSpringer New York LLC; 2015 [cited 13 March 2018].

153. Durojaiye AB, McGeorge NM, Puett LL, Stewart D, Fackler JC, Hoonakker PLT, et al. Mapping the Flow of Pediatric Trauma Patients Using Process Mining. Applied clinical informatics. 2018;9(3):654-66.

154. Quintano Neira RA, Hompes BFA, de Vries JG, Mazza BF, Simões de Almeida, Samantha L., Stretton E, et al. Analysis and Optimization of a Sepsis Clinical Pathway Using Process Mining. Business Process Management; Workshops; Cham: Springer International Publishing; 2019.

155. Defossez G, Rollet A, Dameron O, Ingrand P. Temporal representation of care trajectories of cancer patients using data from a regional information system: An application in breast cancer. BMC Med Informatics Decis Mak. 2014;14(1).

156. Lee N, Laine AF, Hu J, Wang F, Sun J, Ebadollahi S. Mining electronic medical records to explore the linkage between healthcare resource utilization and disease severity in diabetic patients. Proceedings - 2011 1st IEEE International Conference on Healthcare Informatics, Imaging and Systems Biology, HISB 2011; 2011 1st IEEE International Conference on Healthcare Informatics, Imaging and Systems Biology, HISB 2011; 26 July 2011 through 29 July 2011; San Jose, CA. ; 2011.

157. Rojas E, Cifuentes A, Burattin A, Munoz-Gama J, Sepúlveda M, Capurro D. Analysis of Emergency Room Episodes Duration Through Process Mining. Business Process Management; Workshops; Cham: Springer International Publishing; 2019.

158. Rojas E, Cifuentes A, Burattin A, Munoz-Gama J, Sepúlveda M, Capurro D. Performance Analysis of Emergency Room Episodes Through Process Mining. International Journal of Environmental Research and Public Health. 2019;16(7).

159. Rismanchian F, Lee YH. Process Mining–Based Method of Designing and Optimizing the Layouts of Emergency Departments in Hospitals. Health Environ Res Des J. 2017;10(4):105-20.

160. Rojas E, Sepúlveda M, Munoz-Gama J, Capurro D, Traver V, Fernandez-Llatas C. Question-driven methodology for analyzing emergency room processes using process mining. Appl Sci. 2017;7(3).

161. Zhang Y, Padman R. Data-driven clinical and cost pathways for chronic care delivery. Am J Managed Care. 2016;22(12):816-20.

162. Helmering P, Harrison P, Iyer V, Kabra A, Van Slette J. Process mining of clinical workflows for quality and process improvement. Mercy Health System. 2012:1-7.

163. Hilton RP, Serban N, Zheng RY. Uncovering longitudinal healthcare utilization from patient-level medical claims data. arXiv preprint arXiv:1603.00896. 2016.

164. Hirano S, Tsumoto S. Visualizing dynamics of patients in hospitals using devise locations. Conference Proceedings - IEEE International Conference on Systems, Man and Cybernetics; 2014 IEEE International Conference on Systems, Man, and Cybernetics, SMC 2014; 5 October 2014 through 8 October 2014; Institute of Electrical and Electronics Engineers Inc.; 2014.

165. Hirano S, Tsumoto S. Visualization of patient distributions in a hospital based on the clinical actions stored in EHR. IEEE International Conference on Data Mining Workshops, ICDMW; 14th IEEE International Conference on Data Mining Workshops, ICDMW 2014; 14 December 2014; IEEE Computer Society; 2015.

166. Egho E, Jay N, Raïssi C, Nuemi G, Quantin C, Napoli A. An approach for mining care trajectories for chronic diseases. Murcia edSpringer Verlag; 2013 [cited 20 February 2018].

167. Dahlin S, Raharjo H. Relationship between patient costs and patient pathways. Int J Health Care Qual Assur. 2019;32(1):246-61.

168. Arnolds IV, Gartner D. Improving hospital layout planning through clinical pathway mining. Ann Oper Res. 2017:1-25.

169. Mans R, Schonenberg H, Leonardi G, Panzarasa S, Cavallini A, Quaglini S, et al. Process mining techniques: An application to stroke care. Studies in Health Technology and Informatics; ; 2008.

170. Yoo S, Cho M, Kim E, Kim S, Sim Y, Yoo D, et al. Assessment of hospital processes using a process mining technique: Outpatient process analysis at a tertiary hospital. Int J Med Inf. 2016;88:34-43.

171. Halonen R, Martikainen O, Räsänen S, Uusi-Pietila M. Improved Dental Services With Process Modelling. The 11th Mediterranean Conference on Information Systems (MCIS), Genoa, Italy; ; 2017.

172. Partington A, Wynn M, Suriadi S, Ouyang C, Karnon J. Process Mining for Clinical Processes: A Comparative Analysis of Four Australian Hospitals. ACM Trans.Manage.Inf.Syst. 2015 jan;5(4):19:1,19:18.

173. Suriadi S, Mans RS, Wynn MT, Partington A, Karnon J. Measuring patient flow variations: A cross-organisational process mining approach. Brisbane, QLD edSpringer Verlag; 2014 [cited 20 February 2018].

174. Meng F, Ooi CK, Soh CKK, Teow KL, Kannapiran P. Quantifying patient flow and utilization with patient flow pathway and diagnosis of an emergency department in Singapore. Health Systems. 2016;5(2):140-8.

175. Nuemi G, Afonso F, Roussot A, Billard L, Cottenet J, Combier E, et al. Classification of hospital pathways in the management of cancer: Application to lung cancer in the region of burgundy. Cancer Epidemiol. 2013;37(5):688-96.

176. Schwarz K, Römer M, Mellouli T. A data-driven hierarchical MILP approach for scheduling clinical pathways: a real-world case study from a German university hospital. Business Research. 2019;12(2):597-636.

177. Senderovich A, Rogge-Solti A, Gal A, Mendling J, Mandelbaum A, Kadish S, et al. Data-driven performance analysis of scheduled processesSpringer Verlag; 2015 [cited 15 March 2018].

178. Senderovich A, Weidlich M, Gal A, Mandelbaum A, Kadish S, Bunnell CA. Discovery and validation of queueing networks in scheduled processesSpringer Verlag; 2015 [cited 20 February 2018].

179. Senderovich A, Weidlich M, Yedidsion L, Gal A, Mandelbaum A, Kadish S, et al. Conformance checking and performance improvement in scheduled processes: A queueing-network perspective. Inf Syst. 2016;62:185-206.

180. Vahdat V, Namin A, Azghandi R, Griffin J. Improving patient timeliness of care through efficient outpatient clinic layout design using data-driven simulation and optimisation. Health Systems. 2019;8(3):162-83.

181. Andrews R, Wynn MT, Vallmuur K, ter Hofstede, Arthur H. M., Bosley E, Elcock M, et al. Pre-hospital Retrieval and Transport of Road Trauma Patients in Queensland. Business Process Management; Workshops; Cham: Springer International Publishing; 2019.

182. Andrews R, Wynn TM, Vallmuur K, ter Hofstede, H. M. Arthur, Bosley E, Elcock M, et al. Leveraging Data Quality to Better Prepare for Process Mining: An Approach Illustrated Through Analysing Road Trauma Pre-Hospital Retrieval and Transport Processes in Queensland. International Journal of Environmental Research and Public Health. 2019;16(7).

183. Funkner AA, Yakovlev AN, Kovalchuk SV. Towards evolutionary discovery of typical clinical pathways in electronic health records. ; 2017.

184. Klimov D, Shahar Y, Taieb-Maimon M. Intelligent visualization and exploration of time-oriented data of multiple patients. Artificial Intelligence in Medicine. 2010;49(1):11-31.

185. Fernandez-Llatas C, Ibanez-Sanchez G, Celda A, Mandingorra J, Aparici-Tortajada L, Martinez-Millana A, et al. Analyzing Medical Emergency Processes with Process Mining: The Stroke Case. International Conference on Business Process Management; Springer; 2018.

186. Benevento E, Aloini D, Squicciarini N, Dulmin R, Mininno V. Queue-based features for dynamic waiting time prediction in emergency department. Measuring Business Excellence. 2019.

187. Canjels KF, Imkamp MSV, Boymans, T. A. E. J., Vanwersch RJB. Unraveling and improving the interorganizational arthrosis care process at Maastricht UMC+: An illustration of an innovative, combined application of data and process mining. CEUR-WS; 2019.

188. Cho M, Kim K, Lim J, Baek H, Kim S, Hwang H, et al. Developing data-driven clinical pathways using electronic health records: The cases of total laparoscopic hysterectomy and rotator cuff tears. International Journal of Medical Informatics. 2020;133:104015.

189. Johnson OA, Dhafari TB, Kurniati A, Fox F, Rojas E. The ClearPath Method for Care Pathway Process Mining and Simulation. International Conference on Business Process Management; Springer; 2018.

190. Gicquel Q, Tvardik N, Bouvry C, Kergourlay I, Bittar A, Segond F, et al. Annotation methods to develop and evaluate an expert system based on natural language processing in electronic medical recordsIOS Press; 2015 [cited 20 February 2018].

191. Shen C-, Jigjidsuren C, Dorjgochoo S, Chen C-, Chen W-, Hsu C-, et al. A data-mining framework for transnational healthcare system. J Med Syst. 2012;36(4):2565-75.

192. Huang Z, Ge Z, Dong W, He K, Duan H. Probabilistic modeling personalized treatment pathways using electronic health records. Journal of Biomedical Informatics. 2018;86:33-48.

193. Li C, Rana S, Phung D, Venkatesh S. Hierarchical Bayesian nonparametric models for knowledge discovery from electronic medical records. Knowl Based Syst. 2016;99:168-82.

194. Dewandono RD, Fauzan R, Sarno R, Sidiq M. Ontology and process mining for diabetic medical treatment sequencing. Proceedings of The 7th International Conference on Information & Communication Technology and Systems (ICTS); ; 2013.

195. Mans R, Reijers H, Wismeijer D, van Genuchten M. A process-oriented methodology for evaluating the impact of IT: A proposal and an application in healthcare. Information Systems. 2013;38(8):1097-115.

196. Liu H, Li X, Yu Y, Mei J, Xie G, Perer A, et al. Synthesizing Analytic Evidence to Refine Care Pathways. Studies in Health Technology and Informatics; 26th Medical Informatics in Europe Conference, MIE 2015; 27 May 2015 through 29 May 2015; IOS Press; 2015.

197. Meier J, Dietz A, Boehm A, Neumuth T. Predicting treatment process steps from events. J Biomed Informatics. 2015;53:308-19.

198. Kim E, Kim S, Song M, Kim S, Yoo D, Hwang H, et al. Discovery of outpatient care process of a tertiary university hospital using process mining. Healthc Informatics Res. 2013;19(1):42-9.

199. Huang Z, Gan C, Lu X, Huan H. Mining the changes of medical behaviors for clinical pathways. Studies in Health Technology and Informatics; 14th World Congress on Medical and Health Informatics, MEDINFO 2013; 20 August 2013 through 23 August 2013; Copenhagen. ; 2013.

200. Yampaka T, Chongstitvatana P. An application of process mining for queueing system in health service. 2016 13th International Joint Conference on Computer Science and Software Engineering, JCSSE 2016; 13th International Joint Conference on Computer Science and Software Engineering, JCSSE 2016; 13 July 2016 through 15 July 2016; Institute of Electrical and Electronics Engineers Inc.; 2016.

201. Wang T, Tian X, Yu M, Qi X, Yang L. Stage division and pattern discovery of complex patient care processes. J Syst Sci Complex. 2017;30(5):1136-59.

202. Mohammed O, Benlamri R. Developing a Semantic Web Model for Medical Differential Diagnosis Recommendation. J Med Syst. 2014;38(10).

203. Stefanini A, Aloini D, Benevento E, Dulmin R, Mininno V. A data-driven methodology for supporting resource planning of health services. Socio-Economic Planning Sciences. 2019:100744.

204. Huang Z, Lu X, Duan H. Using recommendation to support adaptive clinical pathways. J Med Syst. 2012;36(3):1849-60.

205. Uragaki K, Hosaka T, Arahori Y, Kushima M, Yamazaki T, Araki K, et al. Sequential pattern mining on electronic medical records with handling time intervals and the efficacy of medicines. Proceedings - IEEE Symposium on Computers and Communications; 2016 IEEE Symposium on Computers and Communication, ISCC 2016; 27 June 2016 through 1 July 2016; Institute of Electrical and Electronics Engineers Inc.; 2016.

206. Yang S, Li J, Tang X, Chen S, Marsic I, Burd RS. Process Mining for Trauma Resuscitation. IEEE Intelligent Informatics Bulletin. 2017;18:15-9.

207. Huang Z, Lu X, Duan H, Fan W. Summarizing clinical pathways from event logs. J Biomed Informatics. 2013;46(1):111-27.

208. Xu X, Jin T, Wei Z, Wang J. Incorporating domain knowledge into clinical goal discovering for clinical pathway mining. 2017 IEEE EMBS International Conference on Biomedical and Health Informatics, BHI 2017; 4th IEEE EMBS International Conference on Biomedical and Health Informatics, BHI 2017; 16 February 2017 through 19 February 2017; Institute of Electrical and Electronics Engineers Inc.; 2017.

209. Hwang S, Wei C, Yang W. Discovery of temporal patterns from process instances. Computers in Industry. 2004;53(3):345-64.

210. Yang S, Zhou M, Chen S, Dong X, Ahmed O, Burd RS, et al. Medical Workflow Modeling Using Alignment-Guided State-Splitting HMM. ; 2017.

211. Kirchner K, Herzberg N, Rogge-Solti A, Weske M. Embedding conformance checking in a process intelligence system in hospital environments. In: Process Support and Knowledge Representation in Health Care. Springer; 2013. p. 126-39.

212. Mans RS, van der Aalst W, Vanwersch R. Process mining in healthcare: opportunities beyond the ordinary. BPM reports. 2013;1326.

213. Wang H-, Zhou T-, Tian L-, Qian Y-, Li J-. Creating hospital-specific customized clinical pathways by applying semantic reasoning to clinical data. J Biomed Informatics. 2014;52:354-63.

214. Yan H, Van Gorp P, Kaymak U, Ji L, Lu X, Chiau CC, et al. Variance analysis in Task-Time matrix clinical pathways. 2017 IEEE EMBS International Conference on Biomedical and Health Informatics, BHI 2017; 4th IEEE EMBS International Conference on Biomedical and Health Informatics, BHI 2017; 16 February 2017 through 19 February 2017; Institute of Electrical and Electronics Engineers Inc.; 2017.

215. Yang W-, Hwang S-. A process-mining framework for the detection of healthcare fraud and abuse. Expert Sys Appl. 2006;31(1):56-8.

216. Zhou M, Yang S, Li X, Lv S, Chen S, Marsic I, et al. Evaluation of trace alignment quality and its application in medical process mining. ; 2017.

217. Guyet T, Happe A, Dauxais Y. Declarative sequential pattern mining of care pathwaysSpringer Verlag; 2017 [cited 15 March 2018].

218. Blum T, Padoy N, Feußner H, Navab N. Workflow mining for visualization and analysis of surgeries. International Journal of Computer Assisted Radiology and Surgery. 2008;3(5):379-86.

219. Adeyemi S, Demir E, Chaussalet T. Towards an evidence-based decision making healthcare system management: Modelling patient pathways to improve clinical outcomes. Decis Support Syst. 2013;55(1):117-25.

220. Du G, Jiang Z, Diao X, Yao Y. Knowledge extraction algorithm for variances handling of CP using integrated hybrid genetic double multi-group cooperative PSO and DPSO. J Med Syst. 2012;36(2):979-94.

221. Du G, Jiang Z, Yao Y, Diao X. Clinical pathways scheduling using hybrid genetic algorithm. J Med Syst. 2013;37(3).

222. Movahedi F, Kormos RL, Lohmueller L, Seese L, Kanwar M, Murali S, et al. Sequential pattern mining of longitudinal adverse events after Left Ventricular Assist Device implant. IEEE J Biomed Health Inform. 2019 Dec 9.

223. Dabek F, Chen J, Garbarino A, Caban JJ. Visualization of longitudinal clinical trajectories using a graph-based approach. ACM International Conference Proceeding Series; 2015 Workshop on Visual Analytics in Healthcare, VAHC 2015; 25 October 2015; Association for Computing Machinery; 2015.

224. Chen Y, Ghosh J, Bejan CA, Gunter CA, Gupta S, Kho A, et al. Building bridges across electronic health record systems through inferred phenotypic topics. J Biomed Informatics. 2015;55:82-93.

225. Huang Z, Dong W, Duan H. A probabilistic topic model for clinical risk stratification from electronic health records. J Biomed Informatics. 2015;58:28-36.

226. Riaño D, López-Vallverdú JA, Tu S. Mining hospital data to learn SDA clinical algorithms. Amsterdam ed; 2008 [cited 25 October 2018].

227. Neumuth T, Jannin P, Schlomberg J, Meixensberger J, Wiedemann P, Burgert O. Analysis of surgical intervention populations using generic surgical process models. International Journal of Computer Assisted Radiology and Surgery. 2011;6(1):59-71.

228. Rojas E, Capurro D. Characterization of Drug Use Patterns Using Process Mining and Temporal Abstraction Digital Phenotyping. Business Process Management; Workshops; Cham: Springer International Publishing; 2019.

229. Williams R, Brown B, Peek N, Buchan I. Making medication data meaningful: Illustrated with hypertensionIOS Press; 2017 [cited 15 March 2018].

230. Chen J, Guo C, Sun L, Lu M. Mining Typical Treatment Duration Patterns for Rational Drug Use from Electronic Medical Records. Journal of Systems Science and Systems Engineering. 2019;28(5):602-20.

231. Chen J, Sun L, Guo C, Wei W, Xie Y. A data-driven framework of typical treatment process extraction and evaluation. Journal of Biomedical Informatics. 2018;83:178-95.

232. Kelleher DC, Jagadeesh CB, Waterhouse LJ, Carter EA, Burd RS. Effect of a Checklist on Advanced Trauma Life Support Workflow Deviations during Trauma Resuscitations without Pre-Arrival Notification. J Am Coll Surg. 2014;218(3):459-66.

233. Dauxais Y, Guyet T, Gross-Amblard D, Happe A. Discriminant chronicles mining: Application to care pathways analyticsSpringer Verlag; 2017 [cited 20 February 2018].

234. Sun W, Shen W, Li X, Cao F, Ni Y, Liu H. Mining information dependency in outpatient encounters for chronic disease care. Studies in Health Technology and Informatics; 14th World Congress on Medical and Health Informatics, MEDINFO 2013; 20 August 2013 through 23 August 2013; Copenhagen. ; 2013.

235. Boytcheva S, Angelova G, Angelov Z, Tcharaktchiev D. Mining clinical events to reveal patterns and sequencesSpringer Verlag; 2016 [cited 13 March 2018].

236. Tóth K, Kósa I, Vathy-Fogarassy Á. Frequent treatment sequence mining from medical databasesIOS Press; 2017 [cited 20 February 2018].

237. McGregor C, Catley C, James A. A process mining driven framework for clinical guideline improvement in critical care. Proceedings of the Learning from Medical Data Streams Workshop. Bled, Slovenia (July 2011); ; 2011.

238. Shknevsky A, Shahar Y, Moskovitch R. Consistent discovery of frequent interval-based temporal patterns in chronic patients’ data. J Biomed Informatics. 2017;75:83-95.

239. Huang Z, Bao Y, Dong W, Lu X, Duan H. Online Treatment Compliance Checking for Clinical Pathways. J Med Syst. 2014;38(10).

240. Neumuth T, Liebmann P, Wiedemann P, Meixensberger J. Surgical Workflow Management Schemata for Cataract Procedures. Process Model-based Design and Validation of Workflow Schemata. Methods Inf Med. 2012;51(5):371-82.

241. Valero-Ramon Z, Fernandez-Llatas C, Martinez-Millana A, Traver V. A Dynamic Behavioral Approach to Nutritional Assessment using Process Mining. ; 2019.

242. Weber P, Backman R, Litchfield I, Lee M. A Process Mining and Text Analysis Approach to Analyse the Extent of Polypharmacy in Medical Prescribing. ; 2018.
